# Population Genetic Diversity in the Australian ‘Seascape’: A Bioregion Approach

**DOI:** 10.1371/journal.pone.0136275

**Published:** 2015-09-16

**Authors:** Lisa C. Pope, Cynthia Riginos, Jennifer Ovenden, Jude Keyse, Simon P. Blomberg

**Affiliations:** 1 School of Biological Sciences, The University of Queensland, Brisbane, Australia; 2 Molecular Fisheries Laboratory, School of Biomedical Sciences, The University of Queensland, Brisbane, Australia; The Australian National University, AUSTRALIA

## Abstract

Genetic diversity within species may promote resilience to environmental change, yet little is known about how such variation is distributed at broad geographic scales. Here we develop a novel Bayesian methodology to analyse multi-species genetic diversity data in order to identify regions of high or low genetic diversity. We apply this method to co-distributed taxa from Australian marine waters. We extracted published summary statistics of population genetic diversity from 118 studies of 101 species and > 1000 populations from the Australian marine economic zone. We analysed these data using two approaches: a linear mixed model for standardised data, and a mixed beta-regression for unstandardised data, within a Bayesian framework. Our beta-regression approach performed better than models using standardised data, based on posterior predictive tests. The best model included region (Integrated Marine and Coastal Regionalisation of Australia (IMCRA) bioregions), latitude and latitude squared. Removing region as an explanatory variable greatly reduced model performance (delta DIC 23.4). Several bioregions were identified as possessing notably high genetic diversity. Genetic diversity increased towards the equator with a ‘hump’ in diversity across the range studied (−9.4 to −43.7°S). Our results suggest that factors correlated with both region and latitude play a role in shaping intra-specific genetic diversity, and that bioregion can be a useful management unit for intra-specific as well as species biodiversity. Our novel statistical model should prove useful for future analyses of within species genetic diversity at broad taxonomic and geographic scales.

## Introduction

Genetic diversity can be considered the most basic level of biodiversity [1,2]. The need to conserve genetic diversity is being increasingly recognised by management agencies globally (e.g. [3]), with growing evidence that populations with higher levels of genetic diversity have greater resilience to changing and unpredictable environments [4,5]. Unlike global patterns of species diversity, which show strong latitudinal clines across a broad range of taxa, (e.g. [6,7]), it is yet to be established whether such patterns are common, or consistent, for within species genetic diversity across taxa (but see [8–14]).

Many factors will influence genetic diversity within populations. Neutral population genetic diversity is expected to be proportional to long-term effective population size and mutation [15], which is influenced by a wide range of factors such as: dispersal [16], breeding system [17], historic population size [18], and present-day abundance (e.g. [19] (nuclear only); [20]). It has been suggested that some of the same factors hypothesised to influence species richness could also influence genetic diversity. Such factors are often correlated with latitude, and include: increased mutation rates or relaxed metabolism constraints caused by higher temperatures (e.g. [21,22]); more stable, older populations [23]; and higher density due to greater food availability/niche diversity (e.g. [24]). As for many other species, marine species richness is strongly correlated with latitude, declining away from the equator in coastal species [7,25]. If there is a correlation between species richness and population genetic diversity, termed the species genetic diversity correlation (SGDC; [12,26]), we expect a decline in genetic diversity away from the equator.

Conversely, recent work has demonstrated that marine community evenness (number of individuals of each species) decreased towards the equator due to the presence of a greater number of rare species [27]. We might therefore predict a weaker relationship, or even a reduction in average intra-specific genetic diversity towards the equator due to greater variance in population size. Alternatively, differences among species in dispersal characteristics (e.g. [28]), life history strategy [29], or differences among populations due to factors such as range position (central vs edge populations, e.g. [30]), may not have strong geographic patterns, making concordant geographic patterns in population genetic diversity uncommon (e.g. [11]).

Bioregions represent an attempt to delineate areas containing distinct species assemblages, common across a broad range of taxa. Such regions are likely to possess similarities in both evolutionary history and environmental characteristics, making them a useful unit for testing biogeographic hypotheses [31]. Common evolutionary processes may result in broad-scale patterns of genetic diversity [32], and regions may therefore be more informative than latitude for describing intra-specific diversity. Extensive effort has been placed into designating bioregions within the Australian marine environment, based on the distributions of fish and other species, and bathymetric and environmental variables [33]. This regional framework, the Integrated Marine and Coastal Regionalisation of Australia (IMCRA), has been used to determine representative regions for conservation and management around Australia. However, the relevance of these regions to the distribution of intra-specific genetic diversity is unknown.

In order to determine within species genetic diversity patterns at a broad geographic scale, large amounts of data are required. A synthetic analysis that makes use of existing data from previously published studies has many advantages. Most notably, information can be obtained for a large number of species across a broad geographic extent, for very little cost and low effort. Without such a comprehensive data set, the description of ecosystem scale patterns is compromised. Combining data across multiple studies involves substantial challenges that need to be recognised. These include: combining data from different genetic markers and measures of genetic diversity, uneven geographic sampling, and uneven taxonomic sampling. However, the benefit of being able to generalise across a broad range of species and geographic extent should outweigh these disadvantages, where care is taken in the analysis (see [34–37]).

Australia’s marine exclusive economic zone (EEZ) is one of the most biodiverse regions on earth [38]. There has been significant progress towards cataloguing Australian marine species diversity in recent years (e.g. fishmap, Atlas of Living Australia, http://www.ala.org.au; [39]), however there remain large numbers of species and ecosystems about which little is known [39]. Identification of poorly studied regions is considered a research priority [40]. Here, we perform an analysis of a random sample of published population genetic statistics from marine species around Australia. The majority of these studies provide estimates of neutral population genetic diversity, as few studies to date have estimated functional genetic diversity. We develop a methodology to analyse multi-species genetic diversity data, and use this to identify regions of high/low genetic diversity. We use this information to suggest priorities for future population genetic research in Australian waters. In addition, we test whether there are common patterns of within species genetic diversity that correlate with bioregion or latitude

## Materials and Methods

### Database construction

We performed a literature search on 11^th^ April 2012. We combined searches from five databases: Web of Science, Zoological Record, BIOSIS (Thomson Reuters), Scopus (Elsevier), and ASFA1 (Proquest). Details of these searches are included in S1 Appendix.

The following selection criteria were then applied:

1. Marine organism with at least three populations sampled from the Australian EEZ
2. Minimum of five samples per population
3. Study extent greater than 20 km
4. Information on population H_E_ (expected heterozygosity, [41]), or *h* (haplotype diversity, [42])
5. Some information on the population geographic locations, either through coordinates, place names or clear placement in a figure allowing approximate locations to be derived using Google Earth v7.1.2.2041 (http://www.google.com/earth/).
6. Studies that potentially included multiple cryptic species or hybridization, as indicated by the primary literature, were discarded.

Studies that met these criteria were entered into a database, with each species/marker combination per paper given a unique ‘dataID’. Species were divided into ‘broad taxonomic units’ (BTUs), generally based on Class but sometimes Phylum or Order where there were insufficient species to separate Classes (see Table 1). Genetic markers were assigned to six categories: mitochondrial (mtDNA) sequence, mtDNA restriction enzyme, allozyme, microsatellite, nuclear sequence, other (which included nuclear and mitochondrial single-strand conformation polymorphism (SSCP), inter-simple sequence repeat (ISSR), and denaturing gradient gel electrophoresis (DGGE) studies).

**Table 1:** The number of populations and species included in this regional analysis of genetic diversity, relative to the number of known Australian species. BTU represents a broad taxonomic unit, # pops, # species and % sp. represent the data used in present study (%). Known Aus. species represents the number of species in public databases as reported by [39], presented both as total number (#) and as a % of the total recorded marine species for the Australian economic zone (%). Studied (%) is the percentage of recorded Australian species used in the present study. The table does not include all major marine groups in Australia, but lists the three largest groups not studied.

BTU		This study			Known Aus. species		Studied
		# pops	# species	% sp.	#	%	%
Pisces	Actinopterygii	346	41	48.5%	5184	15.8%	0.9%
	Chondricthyes	46	8				
Cnidaria	Anthozoa	198	10	10.9%	1754	5.3%	0.6%
	Scyphozoa	14	1				
Mollusca	Gastropoda	109	4	8.9%	8525	25.9%	0.1%
	Bivalvia	32	3				
	Cephalopoda	8	2				
Mammals	Mammalia	95	6	5.9%	59	0.2%	10.2%
Echinoderms	Asteroidea	63	4	8.9%	1594	4.8%	0.6%
	Holothuroidea	39	3				
	Echinoidea	29	2				
Crustacea	Malacostraca	56	5	5.9%	6365	19.3%	0.1%
	Brachiopod	3	1				
Reptiles	Reptilia	33	2	2.0%	48	0.1%	4.2%
Plant like	Phaeophyceae	27	2	5.0%	1320	4.0%	0.4%
	Rhodophyceae	16	1				
	Angiosperm	14	2				
Protista	Granuloreticulosea	12	1	1.0%	645	2.0%	0.2%
Aves	Aves	7	1	1.0%	158	0.5%	0.6%
Porifera	Calcarea	4	1	1.0%	1701	5.2%	0.1%
Tunicates	Ascidiacea	3	1	1.0%	866	2.6%	0.1%
Annelida					1558	4.7%	
Platyhelminthes					536	1.6%	
Bryozoans					1062	3.2%	
Total		1154	101		~ 32897		~ 0.3%

We attempted to extract all population genetic studies performed in Australian marine waters by our literature search, though inevitably some studies were missed. While our sample is not exhaustive, it should be unbiased both taxonomically and geographically, and so should reflect genuine taxonomic and geographic sampling biases. To gain an idea as to the proportion of marine population genetic studies undertaken prior to 2012 that were included in our analysis, we performed ad hoc searches of authors that occurred at high frequency in our database, and selected 100 papers. We then determined the proportion of papers identified searching by author that were missing from our original search. We also performed ad hoc searches of under represented taxonomic groups (Platyhelminthes and Annelida). To examine if there has been a change in sampling bias in recent times we implemented the literature search used in mid 2012 on the 20^th^ of January 2015. For the papers that matched our criteria we determined taxonomic group and rough geographic region studied, in order to determine if there had been a major taxonomic or geographic shift in the more recent literature.

### Geographic and taxonomic analyses

The geographic distribution of population samples we obtained was visualised as the number of populations within a radius of 100 km, and density of points per IMCRA region. Maps were produced using ArcMap10.2.2 (ESRI 2011). IMCRA provincial bioregion boundaries (version 4) were obtained from the Atlas of Living Australia website, provided by the Environmental Resources Information Network (c) Commonwealth of Australia, Australian Government Department of the Environment and Heritage 2006).

To assess species similarity among regions we performed a community-style cluster analysis using sampling region as the group of interest and sampled species as presence/absence information, as performed by Keyse et al. [43] (‘region co-sampling cluster analysis’). We calculated Euclidean distances among sites using the vegan package for R ([44,45]), and clustered them into groups using Ward’s Minimum Variance criterion, with input distances squared and output branch lengths square rooted, as recommended by Murtagh and Legendre [46]. Only regions with at least five species sampled were considered.

### Estimating regional mean genetic diversity

A common approach to comparing within species diversity is to standardise data in some way (e.g. [11,47]). While this does allow direct comparison, is has several potential disadvantages. Standardised data will contain much greater ‘noise’ due to the difficulty in obtaining accurate species means, and may be less influenced by environmental gradients due to the restricted geographic distribution of many species and the limitation that all species are assigned the same mean genetic diversity. Given these restrictions, we developed and tested an alternative method to analyse unstandardised population genetic diversity data, and compared this to the standard method. We did this using a Bayesian approach, which is particularly useful when applied to complex problems with no exact analytical solution, and which can be difficult to solve using standard ‘frequentist’ techniques [48]. Due to low sample sizes in most offshore regions, which caused instability in our models, we performed this analysis for coastal regions only.

#### Model construction

To allow a more direct comparison, both analysis methods 1) ‘standardised’ (Z) and 2) ‘unstandardised’ (H), were executed within a Bayesian framework using the software WinBUGS version 1.4.3 [49], called from R using the library R2WinBUGS [50]. The response variable, population genetic diversity, was measured as either haplotype diversity (h; ie. mtDNA) or expected heterozygosity (HE; i.e. nuclear DNA; [41]). ‘Standardised’ models used ‘z-scored’ population genetic diversity as the response variable. These were calculated for each dataID (study:genetic marker combination per paper) by subtracting the mean and dividing by the standard deviation for that dataID group. In the ‘unstandardised’ models, the response variable was raw population genetic diversity. A logit link function was used to link population genetic diversity to the explanatory variables. We fitted linear mixed models to the standardised data, and mixed beta regression models to the unstandardised data. Beta regression is appropriate where the response variable is bounded by 0−1, is continuous and is highly skewed, as is the case with unstandardised population genetic diversity data [51].

We tested the utility of all combinations of five explanatory variables for each analysis type. These variables were: sample size (n), marker (M), species (S), latitude (L), latitude squared (L2) and region (R). Sample size was included as an explanatory term for the dispersion of our measure of genetic diversity (following [20,52]). Genetic marker type (M) was included as a factor with six levels. To attempt to account for similarities in genetic diversity among closely related species, and to weight each species equally, we constructed a correlation matrix from the branch lengths of a taxonomic ‘tree’, based on membership to Class, Order, Family, Genus and species, constructed using Grafen’s [53] branch lengths, with the R package ape and assuming a Brownian motion model of evolution [54]. The matrix was used as a multivariate normal prior for the species effect (S). We assessed both a linear and squared relationship between population genetic diversity and latitude, as covariates. Regional effects can be spatially autocorrelated if the factors causing high or low genetic diversity operate at a broader scale than the selected regions [55]. Potential spatial autocorrelation among IMCRA regions was taken into account using a conditional autoregression (CAR) prior [56]. Adjoining regions were identified and a distance matrix based on the extent of sharing of regional boundaries was constructed using WinBUGS [49].

The model for standardized data (Z, linear mixed effects model) can be formally written as:

$$Z_{i}∼dnorm(m_{i},tau)$$

$$m_{i}=X_{i}\beta \;+R_{Ri}+\;S_{Si}$$

The model for unstandardized data (H, beta–regression model) can be written as:

$$H∼Beta(a_{i},b_{i})$$

$$a_{i}=\;\mu_{i}\delta$$

$$b_{i}=(1-\mu_{i})\delta$$

$$logit(\mu_{i})=X_{i}\beta +\;R_{Ri}+S_{SI}$$

where Xi contains the marker matrix, and latitude variables where appropriate, R is the region effects (with _Ri_ CAR prior) and S is the species effect (with _Si_ multivariate Normal prior, with zero mean and correlation matrix derived from the ‘taxonomic tree’). For further details please see S2 Appendix.

#### Model selection and checking

All models were assessed based on: 1) Deviance Information Criteria score (DIC; [57]) and 2) p-values from posterior predictive tests (PPP). The DIC was calculated within WinBUGS. DIC weights were determined as calculated for AIC weights [58]. We used posterior predictive p values as a form of model checking [59]. P values from posterior predictive tests were determined by using our models to generate expected population genetic diversity values, and comparing these to the observed data [60]. Extreme p values (e.g p < 0.05, p > 0.95) indicate a contradiction of the model by the observed data [61]. We also performed sensitivity tests to determine the influence of various priors, and chose those that gave the lowest DIC scores. Model test runs were performed using three chains for 100 000 iterations, thinning every 100 iterations and with a burnin of 5000. All models showed high levels of convergence based on low autocorrelation, Brooks-Gelman-Rubin scores [62] and mixing profiles of chain traces after this number of iterations.

Once the best model was determined (based on DIC and PPP results), a final run of 500 000 iterations, with thinning every 100 iterations and a burnin of 50 000 was performed. We report the mean as a measure of central tendency and 95% central posterior density intervals for regional mean genetic diversity and latitude effects where appropriate. Models were run for data from coastal regions only, ensuring all data sets used had a minimum of 3 populations once outer regions were excluded. To assess variation in geographic patterns between different taxonomic groups, in addition to the coastal region data set containing all species (959 populations, 84 species), models were also run for a dataset comprised of Actinopterygii and Chondrichthyes (ray-finned and cartilaginous fish) (299 populations, 33 species), and Mollusca (148 populations, 9 species), separately.

## Results

From 118 articles in the primary literature that met our criteria, we obtained 153 data records (each given a unique data ID; multiple dataIDs from a paper resulted from multiple markers and/or multiple species per article). From these we obtained 1154 population data points containing either H_E_ or *h* from 101 species (S1 Table). Coastal regions contained data from 84 species, comprising 959 populations. The percentage of information gained from the six different marker categories was as follows: allozyme 32.8%; microsatellite 31.4%; mtDNA sequence 23.5%; mtDNA restriction enzyme 8.0%; nuclear sequence 1.2%; other 2.9%. The average genetic diversity per marker category is shown in S2 Fig.

From 100 papers identified through an ad hoc search by author, 76 were already captured in our database, suggesting we may have missed 24% of relevant literature in the time period we surveyed. Additional ad hoc searches of taxonomic groups that were under represented in our database (Platyhelminthes and Annelida) did not yield any new articles. Our search of articles from mid 2012 to the 20^th^ of January 2015 detected 978 new publications, of which 57 matched our criteria. These included major taxonomic groups in similar proportions to those identified previously, with Molluscs (7/57) and Crustaceans (6/57) still under represented based on the number of known species around Australia. However, 25% of studies had sites located in either the Australian Bight or Joseph Bonaparte Gulf, suggesting that progress is being made towards a better understanding of these regions.

### Geographic and taxonomic distribution of population genetic studies

**Fig 1:**
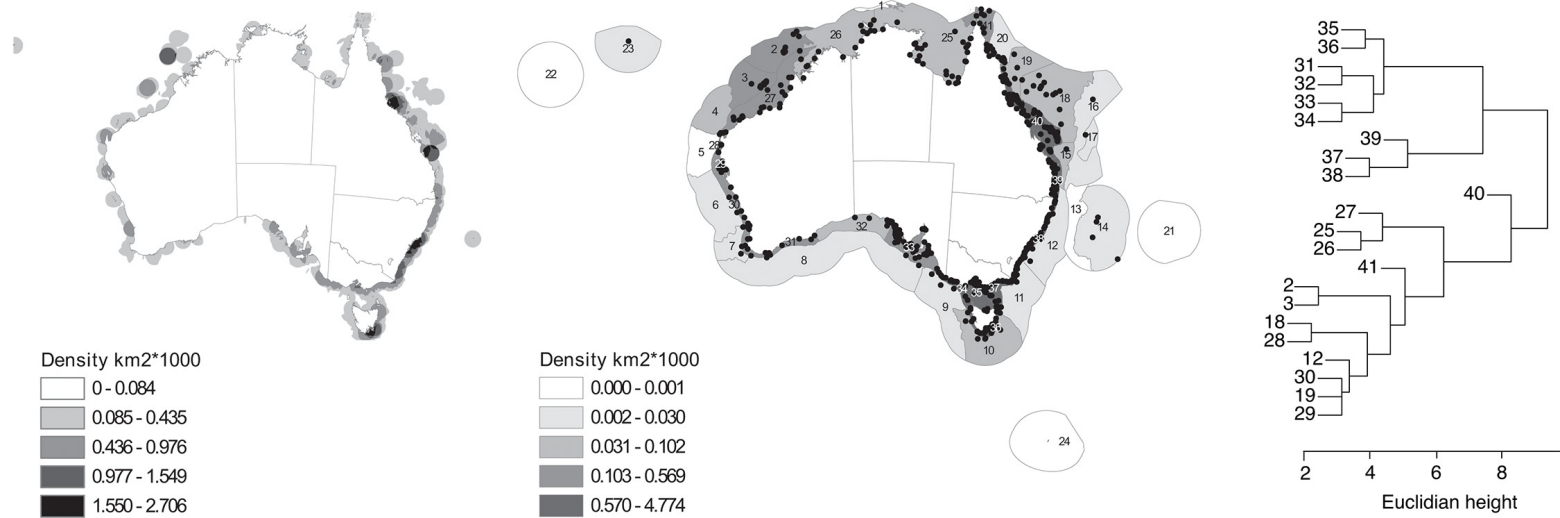
The spatial distribution of populations used to estimate intraspecific genetic diversity for this study. A) The sampling density of populations for estimates of genetic diversity a) over a 100km circle, determined using spatial analyst in ArcMap (ESRI). b) IMCRA region. IMCRA regions are identified by number: regions 1–24 are oceanic or outer shelf, regions 25–41 are coastal and are numbered anti-clockwise starting in the Gulf of Carpentaria. c) A dendrogram of the co-sampling of species among regions, constructed using Euclidean distance. Only regions with at least five species are shown. All maps were produced using GDA94 Australian Albers projection: EPSG 3577.

Sample sites for population genetic studies were not evenly distributed around Australia (Fig 1A and 1B). The majority of sites occurred in coastal areas, particularly the eastern coast. Few of the non-coastal IMCRA regions were well sampled, although four out of 24 non-coastal regions (2, 3, 10, 18) had at least 15 sampling points. The four least studied coastal regions in terms of density were: 26, 32, 25, 27 (Fig 1A and 1B). These included the most northern regions apart from the Great Barrier Reef (GBR): the Gulf of Carpentaria and the ‘Top End’ (25), the Joseph Bonaparte Gulf (26) and northern Western Australia (WA) (27), as well a southern region in the Great Australian Bight (32).

The region co-sampling cluster analysis indicated that neighbouring regions commonly shared surveyed species (Fig 1C). An exception to this was the Great Barrier Reef, regions 40 and 41, which were dissimilar in species inclusion. Region 40 (mid-South GBR) appeared to be rarely co-sampled with any other region, and thus formed a distinct group. There was little co-sampling across the tropical/temperate regions. Regions in the south formed a separate sampling cluster (31−39), with regions along the south-east coast forming a distinct cluster within this group (37−39). There was high co-sampling across the top of Australia (25−27), but less co-sampling across the three ‘tropical’ regions: the ‘Top End’; the GBR; and mid WA (including Ashmore and Scott Reefs, region 2; and Rowley Shoals, region 3).

The taxonomic spread of species targeted for population genetic studies was wide, however most ‘broad operational taxonomic units’ contained small sample sizes (Table 1). The top four taxonomic units based on number of species were: Actinopterygii (41), Anthozoa (10), Chondricthyes (8), Mammalia (6). We compared the species studied in our database to those recorded for the extended Australian marine EEZ ([39]; see Table 1). Groups with a difference of > 5%, which could potentially be considered as well represented in the database included: Pisces, and Cnidirians. Groups with a difference of < 5%, which could be considered to be poorly represented were: Crustacea, and Mollusca (Table 1).

### Patterns of regional marine population genetic diversity

No model for standardised data passed the posterior predictive tests for fit (Table 2, S2 Table). This indicated that none of these models were able to adequately reproduce the observed data. The best model for standardised data that was closest to passing our PPP test (in that the observed value fell closest to the expected distribution) contained sample size and regional effects (SnR). No region had a mean value that was notably different from zero for all standardised models that contained sample size and region, based on 95% central posterior density intervals of regional means, (including the best model). However, standardised models that did not contain sample size (e.g. SR), consistently found that region 25 (Gulf of Carpentaria) had notably high genetic diversity, and region 27 (Nth West coastal) had notably low genetic diversity (6 models).

**Table 2:** Results from models containing increasing numbers of explanatory variables, using both unstandardised population genetic diversity measures (Beta) and standardized genetic diversity (Z) as the response. Explanatory variables are as follows: S = Species, M = genetic marker, L = latitude, L2 = latitude squared, R = IMCRA region, n = sample per population. DIC is the deviance information criterion, and delta represents the difference of a model’s DIC from the best model. PPP is the p value from our posterior predictive tests ([60]) derived from a comparison of the observed data with data generated from the model. Extreme probability values (e.g. p < 0.05, p > 0.95) indicate a contradiction of the model by the observed data [61]. DIC_w_ represents DIC weight calculated according to [58]. The best model from each analysis is indicated with an asterisk.

	Explanatory vars	DIC	delta	DIC_w_	PPP
	Z				
	S + n	2676.3	0.1		>0.0001
	S + n + L	2678.5	2.3		>0.0001
	S + n + L + L2	2680.9	4.7		>0.0001
*	S + n + R	2676.2	0		>0.0001
	S + n + L + R	2677.1	0.9		>0.0001
	S + n + L + L2 + R	2680.2	4.0		>0.0001
	H				
	S + M + n	-661.8	53.7	0	>0.0001
	S + M + n + L	-681.2	34.3	0	0.986
	S + M + n + L + L2	-692.1	23.4	0	0.032
	S + M + n + R	-697.2	18.3	0.01	>0.0001
	S + M + n + L + R	-711.8	3.7	13.6	0.914
*	S + M + n + L + L2 + R	-715.5	0	86.4	0.362

The best overall model, based on both DIC and PPP scores, was the ‘unstandardised’ model that contained species, marker, latitude, latitude squared and regional effects (S + M + n + L + L2 + R; Table 2). This model had a high DIC weight (0.86), indicating it was a great improvement over all other models. The model containing region without latitude (S+M+n+R delta DIC = 18.3), was a better fit than the model with latitude and without region (S+M+n+L+L2 DIC = 23.4), however DIC weight and PPp results indicated that the model containing both region and latitude variables was superior.

**Fig 2:**
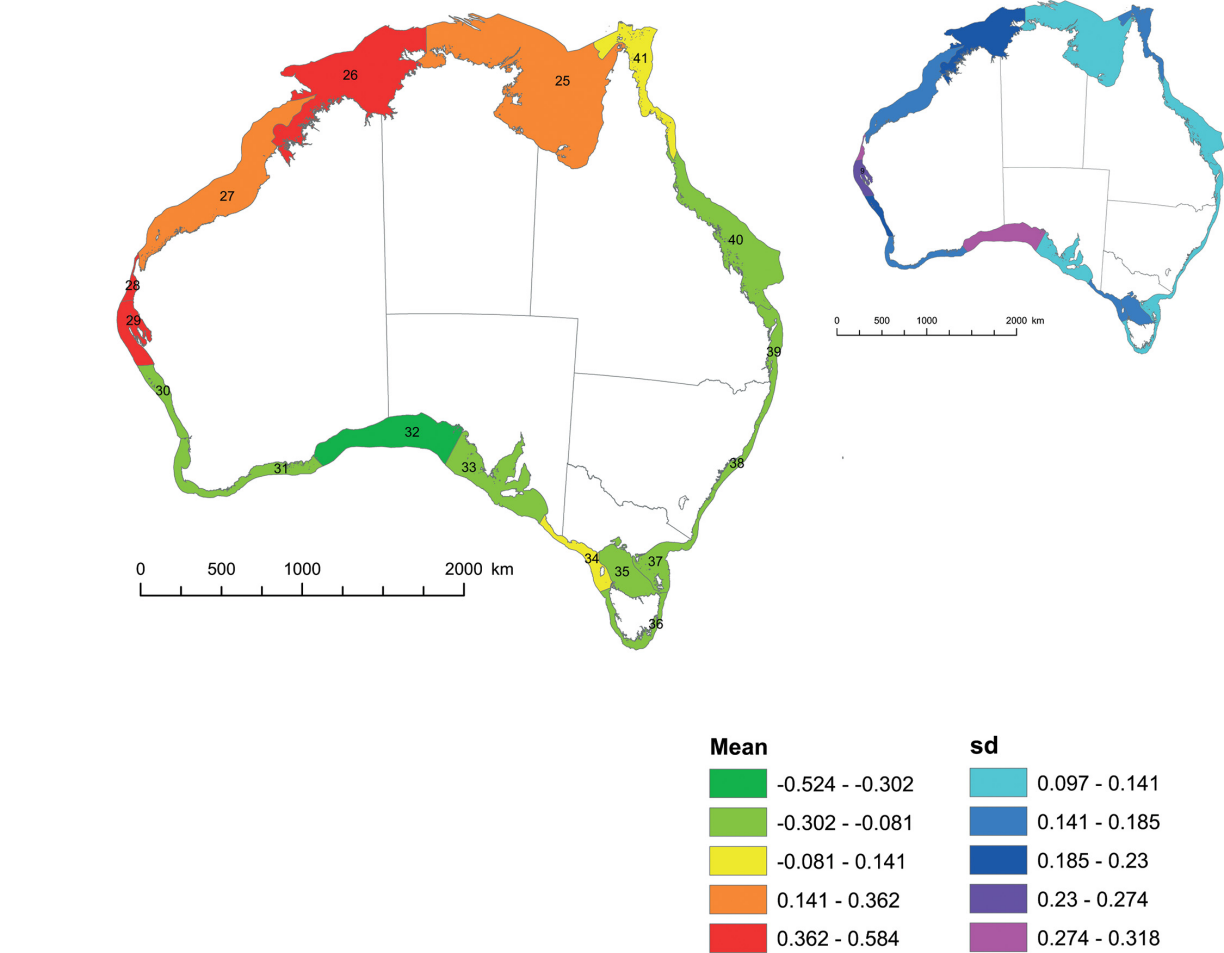
Regional genetic diversity determined using a beta-regression approach with unstandardized population genetic diversity data. The larger map represents regional means, the smaller map the standard deviation within regions. Regions were colour coded by dividing values for each map into five equal intervals; values falling in the upper 20% interval are coloured red/pink, the lowest 20% dark green/light blue etc.

For all unstandardised models where region was included as an explanatory variable, regions 26 (Joseph Bonaparte Gulf) and 29 (Central West coastal) had notably high genetic diversity and region 39 (Southern Qld / N NSW) was notably low (Table 3, Fig 2). In some regional models regions 36 (Tasmania coastal), 31 and 32 (Great Australian Bight) were also notably low. Region 27 (NW coastal) was notably high in the best model only. Where both latitude and latitude squared were included together as explanatory variables, diversity increased towards the equator. Latitude squared had a negative relationship indicating a hump in diversity across the latitudes studied (~ 9.4 to 43.7°S).

**Table 3:** The mean intra population genetic diversity for IMCRA regions (see Fig 2), estimated from models using either standardised (Z) or un-standardised data (H). The best model for standardised genetic data contained a sample size effect, and regional effect in addition to species (SnR). The best model for unstandardized data contained in addition genetic marker, latitude, and latitude squared effects (SnML2R). Results from beta models for fish and molluscs only are also shown. Significant means (those whose MC 95% CI do not span zero) are indicated with an asterisk. Means for regions with less than 10 populations sampled are not shown.

		Z (SnR)				H (SnML^2^R)												
				All		All					Fish			Molluscs				
	IMCRA region	sp.	pop	mean	sd	mean	sd		sp.	pop	mean	sd		sp.	pop	mean	sd	
25	Gulf of Carpentaria	17	52	0.146	0.127	0.249	0.139		9	33	**0.418**	0.221	*			na		
26	Joseph Bonaparte Gulf	11	21	0.138	0.135	**0.584**	0.204	*	7	16	**0.525**	0.270	*	1	2	-2.286	0.626	*
27	NW coastal	17	46	-0.154	0.137	**0.294**	0.144	*	8	18	0.092	0.238		2	11	-2.130	0.437	*
28	CW coastal trans	6	5	-0.089	0.145	0.406	0.283		2	3	0.224	0.272				na		
29	CW coastal	5	17	0.070	0.132	**0.547**	0.259	*	2	2	0.150	0.350				na		
30	SW coastal trans	5	16	-0.005	0.134	-0.172	0.207		3	3	-0.248	0.320				na		
31	SW coastal	11	40	-0.104	0.112	-0.280	0.160		3	11	**-0.547**	0.299	*	1	1	-0.390	0.633	
32	Great Australian Bight	7	15	0.048	0.127	-0.524	0.318		3	3	-0.034	0.332		1	1	0.199	0.565	
33	Spencer Gulf	15	59	-0.062	0.095	-0.125	0.132		4	10	-0.407	0.274		3	13	1.132	0.309	*
34	W Bass Straight	11	41	0.088	0.105	0.126	0.151		4	10	0.095	0.240		2	22	1.768	0.325	*
35	Bass Straight coastal	14	39	-0.056	0.109	-0.181	0.151		7	19	-0.391	0.234		3	10	0.987	0.326	*
36	Tasmania coastal	13	79	-0.069	0.097	-0.246	0.131		6	17	-0.324	0.230		2	50	0.742	0.260	*
37	SE coastal	25	105	0.075	0.095	-0.120	0.115		12	47	0.160	0.191		2	11	0.120	0.269	
38	Central NSW coastal	23	102	0.048	0.080	-0.127	0.116		9	23	0.351	0.228		3	16	-0.060	0.285	
39	Southern Qld / N NSW	25	67	-0.053	0.093	**-0.268**	0.130	*	11	22	-0.041	0.190		2	7	-0.241	0.366	
40	Southern GBR	47	212	-0.010	0.072	-0.136	0.097		23	58	-0.230	0.184		2	3	0.018	0.382	
41	Northern GBR	18	43	-0.011	0.097	-0.027	0.146		4	4	0.208	0.288		1	1	0.140	0.526	
	Latitude			na		0.558	0.319		33	299	0.402	0.579		9	148	-0.043	0.481	
	Total / Latitude^2^	84	959	na		**-0.920**	0.224	*			-0.323	0.467				-0.055	0.360	

There was little correspondence between regional means estimated using the standardised approach, compared to the unstandardised approach based on correlations between regional means (R^2^ = 0.02, p = 0.26)(Table 3, S1 Fig). Focusing on the unstandardised approach, regional means for ray-finned and cartilaginous fish were similar to those found for the entire data set (for regions where at least 10 ‘fish’ populations were sampled (n = 12, R^2^ = 0.51, p = 0.005). However, when comparing regions where Molluscs were sampled with at least 10 populations there was no relationship between the entire dataset and Molluscs (n = 7, R^2^ = 0.14, p = 0.22; S1 Fig).

## Discussion

This study represents the largest synthesis to date of marine population genetic studies at an Australia wide scale. We have developed statistical methods to allow the regional analysis of population genetic data from multiple sources. This well parameterised Bayesian method appears to be an improvement over the more commonly used ‘standardisation’ approach, based on posterior predictive testing. Region was found to be a useful explanatory variable in describing the broad scale distribution of genetic diversity, in addition to latitude. Genetic diversity increased towards the equator, however the relationship between diversity and latitude was best described as ‘humped’, rather than linear. Our study has identified several regions as having either higher or lower genetic diversity than those around them (particularly high diversity in the Joseph Bonaparte Gulf (IMCRA 26) and low diversity in southern Queensland, northern NSW (IMCRA 39). Describing this regional variation represents a first step towards understanding the broad scale processes that might be driving them.

### Geographic and taxonomic representation

The distribution of information on population genetic diversity was uneven around Australia. Sampling was primarily coastal and highest on the east coast of Australia, particularly the Great Barrier Reef. While well studied, the southern GBR has not often been co-sampled with other regions,. This lack of co-sampling of the GBR with other regions has previously been identified based on a dataset of tropical species spanning the Indo-Pacific [43]. The ‘Top End’, Gulf of Carpentaria, Joseph Bonaparte Gulf and the Great Australian Bight were relatively poorly studied.

There are over 32000 species recorded in major databases as occurring in the Australian marine economic zone, though, based on the rate of discovery of undescribed species, the actual number is far higher [39]. While we attempt to describe regional genetic diversity across a wide range of species, this study captures an extremely small part of recorded species diversity (101 species, 0.3%). The majority of species included in our analyses were ray-finned and cartilaginous fish (48%), which were over-represented, in that only ~16% of recorded Australian marine species are Pisces. Yet despite this <1% of recorded ‘fish’ species were included in our database. Cnidirians (10.9% vs 5.3%) and Mammals (~10% of known species) were also relatively well represented according to the proportions in our database compared to that of known Australian species. On the same basis, Crustacea (5.9% vs 19.3%) and Mollusca (8.9% vs 25.9%) were under represented.

Genetic information, in combination with other methods, is extremely useful for defining biological stocks [63,64], an understanding of which is necessary to achieve sustainable harvesting [65]. Australia is moving towards the management of its commercial marine resources as biological stocks rather than by political boundaries [66], however for many species information is lacking. Of the 49 most important commercial taxa in Australia identified in a National Stock Structure of Australia report [66], only 20% were found to have existing population genetic data that fitted our search categories (as defined in the methods).

### Latitudinal patterns of genetic diversity

Genetic diversity followed the expected negative relationship with latitude, increasing towards the equator. Species richness has a strong correlation with latitude (reviewed in [67,68]), which is in turn correlated with many environmental variables, such as sea surface temperature, and coastal complexity [7]. While not directly tested here, as species richness is correlated with latitude, our results provide some support for the ‘species genetic diversity correlation’ in the marine environment [12,26]. However, models with a non-linear relationship between latitude and genetic diversity were a better fit than a simple linear relationship. A ‘humped’ relationship between genetic diversity and latitude has previously been reported in a study of tropical birds [10] where it was suggested this pattern might be caused by reduced diversity at range edges occurring over both ecological (see [69]) and evolutionary (i.e. geometric contraints; [70]) time frames. This argument requires congruence in the range edge of many species, and/or that range edge effects are extensive, and thus may only apply to certain regions and species groups.

### Regional genetic diversity

IMCRA region was a useful concept to explain the distribution of intra-specific genetic variation, both for standardised and unstandardised data. Regional patterns persisted after latitude had been accounted for, and so additional explanations for these differences are needed. Three regions were found to possess ‘high’ levels of genetic diversity (The Joseph Bonaparte Gulf (26), NW coast (27) and the Central West coast (29)), and one possessed ‘low’ diversity (Queensland/northern NSW (39), based on the best fitting unstandardised model.

Areas of high diversity might represent regions of admixture, where distinct evolutionarily significant units overlap (e.g. ‘centre of overlap’, [71]). High within species genetic divergence has been reported either side of Torres Strait (e.g. [72]), which was impassable at times of low sea level, and disparate species groups have been reported for some species along the western coast [73–75]. However, neither high diversity region is exactly congruent with these potential ‘admixture zones’. A related explanation for the low diversity observed in Sth Queensland/Nth NSW (IMCRA 39) may be the presence of large numbers of range edge populations, potentially lowering average intra-specific genetic diversity. However, meta-analyses suggest that range edge populations do not always contain lower diversity [30], weakening this argument. Now that patterns of regional intra-specific genetic diversity have been described, further testing of their generality can take place, and hypotheses to explain their origin can be generated and tested.

### Limitations of our approach

It was surprisingly difficult to limit a search to the marine environment in a specific geographic area, despite attempting to do so across five different databases. While we may have extracted data from approximately 75% of relevant publications, it is unlikely that there was a strong taxonomic bias in this missing data due to our search methodology. It is possible that there was some geographic bias, as some databases required the use of place names to restrict to location. However, as we included the terms ‘Joseph Bonaparte Gulf’ and ‘Australian Bight’ we believe our finding that these regions were under sampled remains valid. Our survey of recent literature suggests that while taxonomic biases remain, some progress is being made towards a better understanding of the Great Australian Bight and Joseph Bonaparte Gulf. We hope that this first attempt to summarise population genetic research in the Australian marine environment will provide a useful starting point for future researchers.

The majority of population genetic analyses performed on wild species to date have utilised neutral genetic markers, due to their greater tractability for analyses. It may be that for conservation purposes, functional genetic diversity is of greater relevance, as this diversity will result in a change in an organism’s phenotype, however there generally appears to be weak correlation between functional and neutral genetic diversity [76,77]. Furthermore, while our focus here in on average population genetic diversity, with the idea that high diversity populations are of higher priority for conservation, it has also been argued that a suite of populations should be selected based on the overall proportion of the species’ genetic diversity that they represent, potentially prioritising populations that have low, but unique, genetic diversity [78,79]. It was not possible for us to consider population ‘unique’ diversity, or functional genetic diversity with the summary data we obtained. Future research may combine both these approaches across multiple species to assist in identification of priority regions for conservation.

The majority of studies considering broad patterns of population genetic diversity, by necessity, focus on common, widely distributed species (e.g. [9]). Rare species may possess lower levels of genetic diversity due to lower effective population size. Our likely bias towards common species may have inflated regional means in tropical areas, where a greater proportion of rare species occur [27]. Thus, the possibility remains that a different relationship between latitude and ‘average’ intraspecific genetic diversity could be observed if the sampling of species across regions was a better representation of both the common and rare species present.

Taxonomic groups were not evenly represented across regions and, despite the inclusion of species as an explanatory variable, this taxonomic bias will have influenced our results. This is evidenced by the low regional mean obtained for molluscs from the highest diversity region in the entire and ‘fish’ only data sets (Table 1, S1 Fig). Differences in regional diversity among taxa may result from many factors such as: life history differences [29], dispersal abilities [16], body size [20], and mutation rates [80], all of which often have a taxonomic component. Increasing species numbers, and improving the taxonomic spread would improve results. Depending on the management objectives, selecting particular species subsets may be of greater relevance.

Pelagic marine species richness was shown to peak in the mid latitudes (20–40 degrees), whereas coastal species peaked in the western Pacific, close to the equator [7]. Our study focused on coastal species and regions, as data for outer regions were limited. Pelagic species may show different regional trends to those we describe for coastal species, and more information on these species might provide greater insight into evolutionary processes.

We focus on regional means, and do find some significant results, yet the large standard deviations we obtained indicate extensive variation exists within regions (Table 3, Fig 2). While our results support the occurrence of general patterns [12], given the preceding caveats, we acknowledge that the extent to which these patterns apply across all species remains unsure [11].

### A new statistical model for genetic diversity indices

Posterior predictive testing provided greater support for the unstandardised Bayesian models, indicating that these models were better able to reproduce results comparable to the observed data. It is perhaps not surprising that there was little correspondence between estimates of regional means using the two methods. Using the standardised approach, we removed the effects of marker and species by giving each study the same mean. While this does allow studies to be compared directly, we lose information as to whether some species have higher ‘absolute’ levels of genetic diversity. It is possible that the Bayesian analysis has captured geographic variation in ‘absolute’ genetic diversity to some extent, allowing a broader exploration of geographic variation in intra-specific diversity.

Additionally, to accurately standardise data requires a good understanding of the underlying mean and distribution for the species. For studies with few populations sampled, z-scored data will not be accurate, and this inaccuracy may detract from the underlying signal obtained from better sampled species. This may be the reason our standardised models failed all posterior predictive tests. If sufficient populations were available to calculate a reliable mean and standard deviation for a study, z-score transformed data may provide useful insight into whether areas possess unusually high or low genetic diversity relative to the species as a whole. However, if only smaller sample sizes are available, this may generate so much noise in the data that the results may be of limited use.

While the unstandardised Bayesian approach represents an advance in statistical methods for combining summary statistics calculated from diverse genetic marker types and species, further improvements are possible. If more data were available, a larger number of genetic marker categories might improve model performance. McCusker and Bentzen [20] found substantial differences between genetic diversity estimates derived from mtDNA control region sequences, as compared to other sections of mtDNA, which they attributed to a faster mutation rate. A different measure of population genetic diversity, such as nucleotide diversity, might provide more insight into evolutionary processes. Nucleotide diversity provides information on both the number of haplotypes and how divergent those haplotypes are within a population. Areas of admixture would be highlighted using this type of measure, which may provide greater insight into evolutionary processes. Finally, a phylogenetic correction for the species effect, the inclusion of a larger number of life history traits [29], and factors such as range position (edge/middle) might help explain a greater proportion of the variation observed.

### Management implications and future research priorities

1. Data collected for this analysis suggests that there are gaps in available information for some marine regions around Australia, particularly for most offshore regions, the Great Australian Bight and Gulf of Carpentaria (though this may be improving), and for taxonomic groups such as Crustacea and Mollusca. Analysis of co-sampling of species across regions indicated that species from the southern GBR (region 40) were often not sampled in other regions. Only 20% of Australia’s top 49 commercial marine species were included in this analysis. Expansion of this database would allow these gaps to be confirmed and prioritised for future research.
2. In addition to information on intraspecific genetic diversity, population genetic data on connectivity is often included in population genetic studies, in the form of summary statistics such as F_ST_. While careful statistical analysis would be required to take into account factors such as distance between populations, and population genetic diversity and its influence on estimates of F_ST_, this wealth of information could allow a broad scale insight into connectivity among multiple species around Australia. We advocate public data archiving for marine genetic studies that make population datasets more visible and enable deeper connections between data, regions and study species–including analysis of connectivity [81]. We encourage the use and expansion of the database associated with this paper.
3. Information on average regional genetic diversity could be incorporated into the decision framework for the designation of marine park areas, with the potential for increased protection afforded to areas of consistently high diversity. For example, the area around Joseph Bonaparte Gulf, was identified as possessing high genetic variation, yet this region is under-represented in the existing Commonwealth reserve system [82].
4. Genetic marker systems that assay a larger proportion of the genome are becoming increasingly cost-effective for non-model organisms. Increased use of such datasets, particularly if there was also geographic consistency in sampling locations across species, may provide a more direct avenue towards describing broad scale patterns of population genetic diversity and connectivity in the future by using estimates that are more directly comparable and accurate, and include both functional and neutral estimates of population genetic diversity e.g. [83].

## Supporting Information

S1 Appendix Literature search method. (DOCX)

S2 Appendix Winbug code for the best unstandardised and standardised models. (ZIP)

S3 Appendix Raw data file containing article ID, marker category, individual population heterozygosity values, and xy coordinates. See associated readme.txt for a full explanation of column headings.
(ZIP)

S1 Fig Regional means for standardised data, and for fish and molluscs only, using unstandardised genetic diversity in a Bayesian model. (EPS)

S2 Fig Population heterozygosity or haplotype diversity for each genetic marker category. (TIFF)

S1 Table A list of the species and references from which population genetic diversity data were obtained. (DOCX)

S2 Table Results from all models for both unstandardised (H) and standardized (Z) population genetic diversity measures. (DOCX)
