# Supplementary material for: Population Genetic Diversity in the Australian ‘Seascape’: A Bioregion Approach
Source: PLoS One. 2015 Sep 16;10(9):e0136275. doi: 10.1371/journal.pone.0136275 (PMC4574161; doi:10.1371/journal.pone.0136275)
Supplement: S1 Appendix — (DOCX) [file pone.0136275.s001.docx]

**S1 Appendix** Literature search method

We performed our literature search on 11^th^ April 2012. In order to perform the broadest search possible, we combined searches from five databases: Web of Science, Zoological Record, BIOSIS (Thomson Reuters), Scopus (Elsevier), and ASFA1 (Proquest). For each search we combined three main queries: ‘genetic’, ‘marine’ and ‘Australia’. Details of these are included in the supplementary material. The number of hits from each database for the three search terms combined was as follows: Web of Science 1336, Zoological Records 1593, Biosis 556, Scopus 136, ASFA1 376. After duplicates were removed there were 2881 references imported into Endnote software (Thomson Reuters) from the combined searches. Titles and abstracts of papers were read and those that were clearly irrelevant were discarded.

For all five databases we used the following Boolean combinations: ‘genetic’ (allozyme*, “gene flow”, microsatellite*, “SSRs”, “STRs”, mtDNA , phylogeograph*, “SNP”) combined by AND with ‘marine’ (coral*, estuar*, intertidal, marine, reef, sea, subtidal, ocean*, brackish, mangrove* OR wetland*). We did not include the terms: population structure, biogeograph* or gene as these increased the number of hits dramatically, and for the first two terms resulted in a large number of non-genetic studies.

To limit our search results to the Australian region, we used different strategies depending on the database. For the Web of Science and Scopus search we had to use key words to limit our geographic search. This list of ‘Australia’ key words was combined with the previous search with the AND term: “Pacific ocean”, “Indian ocean”, “Great Barrier Reef”, “Torres Strait”, Australia*, “New Zealand”, Indonesia, Queensland, “Papua New Guinea”, “New South Wales”, “Northern Territory”, “East Timor”, “Gulf of Carpentaria”, “Shark Bay”, “Botany Bay”, Tasmania, “Bass Strait”, “Coral Sea”, “Timor Sea”, “Great Australian Bight”, “Arafura Sea”, “Tasman Sea”, “Southern Ocean”, “Spencer Gulf”, “King Sound”, “Joseph Bonaparte Gulf “, “Hervey Bay”, Ningaloo, “Leeuwin current”, “East Australian current”, “Lord Howe Island”, “Maquarie Island” OR “Port Philip Bay”.

In the other four database searches the ‘Australia’ query could be geographically limited using categories or descriptors. For our Zoological Records search we used the following descriptors: Pacific Ocean, Pacific Ocean Islands, West Pacific, South Pacific, South Indian Ocean or Australia. For the Biosis database we used the geographic tag ‘Australasian region’ to geographically limit our search. For the Scopus database we could limit our search to the exact keywords: "Pacific Ocean”, "Australia" or "Indian Ocean". In the ASFA1 database we were able to refine by location using the following terms: ‘Australia’, ‘ISEW Australia Queensland Great Barrier Reef’, ‘ISW Australia Western Australia’, ‘Australia Coasts’, ‘ISEW Indonesia’, I Pacific, I Indo Pacific, PSE Australia, PSE Australia, NSW.
