# Supplementary material for: Population Genetic Diversity in the Australian ‘Seascape’: A Bioregion Approach
Source: PLoS One. 2015 Sep 16;10(9):e0136275. doi: 10.1371/journal.pone.0136275 (PMC4574161; doi:10.1371/journal.pone.0136275)
Supplement: S3 Appendix — See associated readme.txt for a full explanation of column headings. (ZIP) [file pone.0136275.s003.zip › S3_Appendix _readme.docx]

**S3 Appendix read me file.**

Please see below an explanation for each column heading for raw data file H3. To determine the original reference from which the data was obtained please use Table S1 to cross reference DataID or RefID with the correct citation.

FID_ A unique number for each record
RefID Unique number for each manuscript
DataID Unique number for each species/ marker combination
popname Geographic location name
Region IMCRA region number
Heh Heterozygosity or haplotype diversity
Marker Genetic marker category
n sample size from the population that was genotyped
Genus_spec Genus_species name
Class
Order
Family
Genus
pop_x longitude
pop_y latitude
Ho Observed heterozygosity
He Expected heterozygosity
An Allele number
Ar Allelic richness
h Haplotype diversity
pi Nucleotide diversity
hap Number of haplotypes
Common_name
BTU Broad taxonomic unit
Phylum
Kingdom
Z standardized genetic diversity
