# Supplementary figures and images for: Population Genetic Diversity in the Australian ‘Seascape’: A Bioregion Approach
Source: PLoS One. 2015 Sep 16;10(9):e0136275. doi: 10.1371/journal.pone.0136275 (PMC4574161; doi:10.1371/journal.pone.0136275)

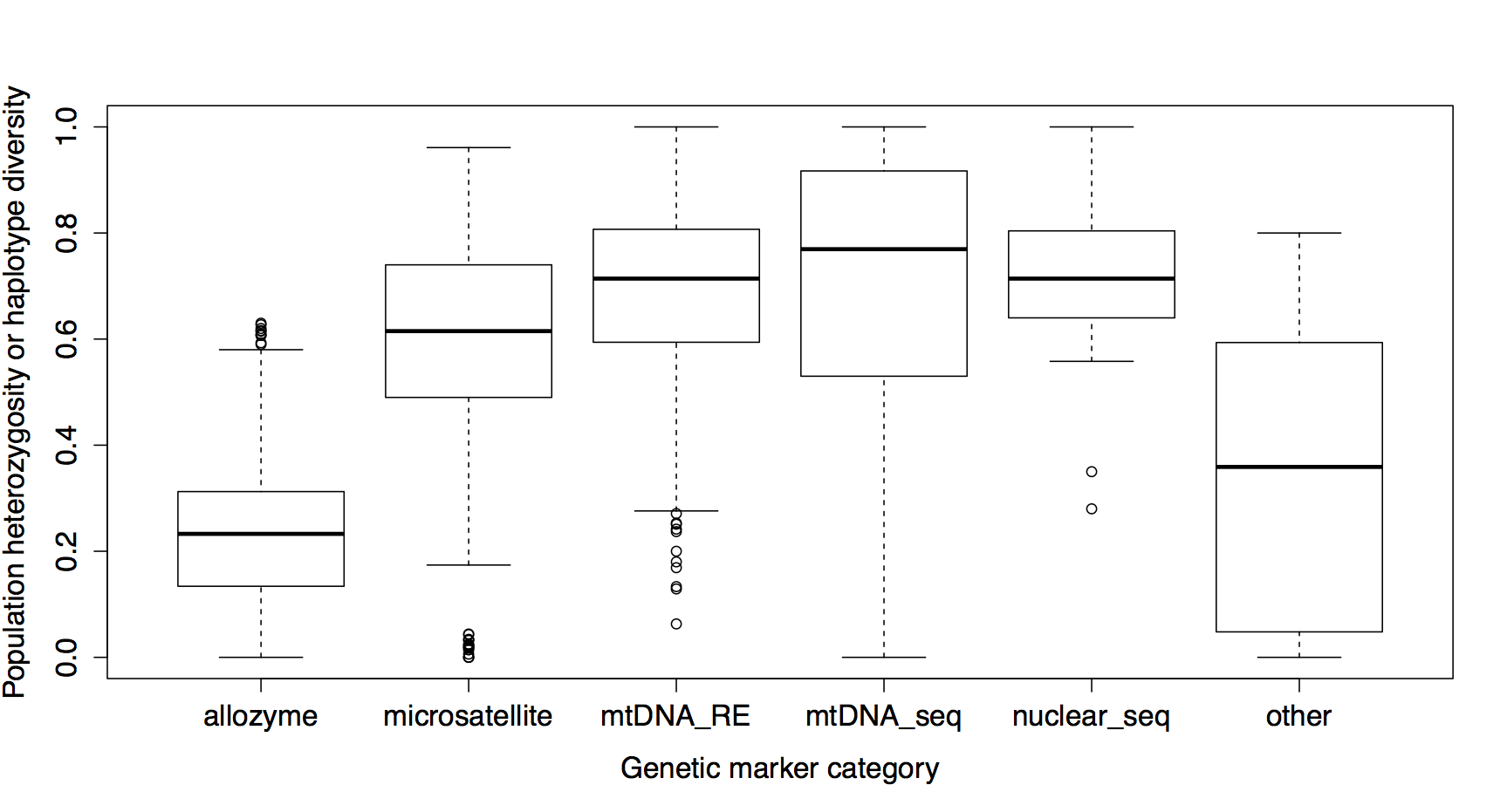

Supplement: S2 Fig — (TIFF) [file pone.0136275.s005.tiff]
