# Supplementary material for: Population Genetic Diversity in the Australian ‘Seascape’: A Bioregion Approach
Source: PLoS One. 2015 Sep 16;10(9):e0136275. doi: 10.1371/journal.pone.0136275 (PMC4574161; doi:10.1371/journal.pone.0136275)
Supplement: S1 Table — (DOCX) [file pone.0136275.s006.docx]

Table S1. A list of the species and references from which population genetic diversity data were obtained. There is a separate line from each manuscript specifying a distinct species marker combination, but each manuscript is only listed once.

| DataID | RefID | Genus species | Marker category | Reference | | | |
| --- | --- | --- | --- | --- | --- | --- | --- |
| 1 | 1 | *Pocillopora damicornis* | allozyme | [Stoddart 1984](#_ENREF_91) | | | |
| 5 | 5 | *Lingula anatina* | allozyme | [Hammond & Poiner 1984](#_ENREF_49) | | | |
| 6 | 6 | *Acanthaster planci* | allozyme | [Nash *et al.* 1988](#_ENREF_73) | | | |
| 8 | 8 | *Marginopora vertebralis* | allozyme | [Benzie 1991](#_ENREF_6) | | | |
| 9 | 9 | *Haliotis rubra* | allozyme | [Brown 1991](#_ENREF_21) | | | |
| 10 | 10 | *Acanthaster planci* | allozyme | [Benzie & Stoddart 1992](#_ENREF_11) | | | |
| 11 | 11 | *Triaenodon obesus* | mtDNA seq | [Whitney *et al.* 2012](#_ENREF_116) | | | |
| 12 | 12 | *Carcharhinus leucas* | mtDNA seq | [Tillett *et al.* 2012b](#_ENREF_97) | | | |
| 18 | 14 | *Glaucosoma hebraicum* | microsatellite | [Berry *et al.* 2012a](#_ENREF_12) | | | |
| 20 | 15 | *Carcharodon carcharias* | mtDNA seq | [Blower *et al.* 2012](#_ENREF_17) | | | |
| 21 | 16 | *Lethrinus nebulosus* | microsatellite | | [Berry *et al.* 2012b](#_ENREF_13) | | |
| 22 | 17 | *Lutjanus carponotatus* | mtDNA seq | [Veilleux *et al.* 2011](#_ENREF_113) | | | |
| 23 | 18 | *Acropora millepora* | microsatellite | [van Oppen *et al.* 2011b](#_ENREF_112) | | | |
| 24 | 19 | *Seriatopora hystrix* | microsatellite | [van Oppen *et al.* 2011a](#_ENREF_111) | | | |
| 27 | 20 | *Rhizoprionodon acutus* | mtDNA seq | [Ovenden *et al.* 2011](#_ENREF_76) | | | |
| 28 | 20 | *Sphyrna lewini* | mtDNA seq |  | | | |
| 29 | 21 | *Carcharhinus amboinensis* | mtDNA seq | [Tillett *et al.* 2012a](#_ENREF_96) | | | |
| 31 | 22 | *Delphinus delphis* | microsatellite | [Moller *et al.* 2011](#_ENREF_71) | | | |
| 32 | 22 | *Delphinus delphis* | mtDNA seq | | | | |
| 33 | 23 | *Apogon doederleini* | mtDNA seq | [Mirams *et al.* 2011](#_ENREF_70) | | | |
| 34 | 23 | *Pomacentrus coelestis* | mtDNA seq |  | | | |
| 35 | 23 | *Dascyllus trimaculatus* | mtDNA seq |  | | | |
| 36 | 23 | *Acanthurus triostegus* | mtDNA seq |  | | | |
| 37 | 24 | *Eleutheronema tetradactylum* | mtDNA seq | [Horne *et al.* 2011](#_ENREF_50) | | | |
| 38 | 24 | *Eleutheronema tetradactylum* | microsatellite |  | | | |
| 39 | 25 | *Scomberomorus commerson* | mtDNA seq | [Sulaiman & Ovenden 2010](#_ENREF_93); [Fauvelot & Borsa 2011](#_ENREF_41) | | | |
| 40 | 26 | *Neophoca cinerea* | mtDNA seq | [Lowther *et al.* 2012](#_ENREF_60) | | | |
| 41 | 27 | *Prototroctes maraena* | microsatellite | [Schmidt *et al.* 2011](#_ENREF_83) | | | |
| 42 | 27 | *Prototroctes maraena* | mtDNA seq |  | | | |
| 45 | 30 | *Scomberomorus semifasciatus* | mtDNA seq | [Broderick *et al.* 2011](#_ENREF_19) | | | |
| 47 | 31 | *Heliocidaris erythrogramma armigera* | microsatellite | [Binks *et al.* 2011](#_ENREF_16) | | | |
| 48 | 32 | *Pinctada maxima* | microsatellite | [Benzie & Smith-Keune 2006](#_ENREF_10) | | | |
| 49 | 33 | *Eubalaena australis* | mtDNA seq | [Carroll *et al.* 2011](#_ENREF_24) | | | |
| 50 | 33 | *Eubalaena australis* | microsatellite |  | | | |
| 53 | 35 | *Acanthopagrus australis* | microsatellite | [Roberts & Ayre 2010](#_ENREF_81) | | | |
| 54 | 36 | *Tursiops aduncas* | other | [Wiszniewski *et al.* 2010](#_ENREF_118) | | | |
| 56 | 37 | *Arctocephalus pusillus doriferus* | mtDNA seq | [Lancaster *et al.* 2010](#_ENREF_58) | | | |
| 59 | 39 | *Phycomenes zostericola* | mtDNA seq | [Haig *et al.* 2010](#_ENREF_48) | | | |
| 64 | 43 | *Plectropomus maculatus* | mtDNA seq | [Evans *et al.* 2010](#_ENREF_40) | | | |
| 65 | 43 | *Lutjanus carponotatus* | mtDNA seq |  | | | |
| 66 | 44 | *Centrostephanus rodgersii* | microsatellite | [Banks *et al.* 2010](#_ENREF_4) | | | |
| 67 | 45 | *Acanthaster planci* | microsatellite | [Yasuda *et al.* 2009](#_ENREF_119) | | | |
| 71 | 47 | *Seriatopora hystrix* | microsatellite | [Bongaerts *et al.* 2010](#_ENREF_18) | | | |
| 73 | 48 | *Plectropomus leopardus* | mtDNA seq | [van Herwerden *et al.* 2006](#_ENREF_109); [van Herwerden *et al.* 2009b](#_ENREF_110) | | | |
| 74 | 49 | *Lethrinus miniatus* | mtDNA seq | [van Herwerden *et al.* 2009a](#_ENREF_107) | | | |
| 75 | 49 | *Lutjanus sebae* | mtDNA seq |  | | | |
| 76 | 50 | *Stegostoma fasciatum* | mtDNA seq | [Dudgeon *et al.* 2009](#_ENREF_38) | | | |
| 77 | 50 | *Stegostoma fasciatum* | microsatellite |  | | | |
| 78 | 51 | *Polydactylus macrochir* | mtDNA seq | [Horne *et al.* 2012](#_ENREF_51) | | | |
| 79 | 52 | *Octopus maorum* | microsatellite | [Doubleday *et al.* 2009](#_ENREF_36) | | | |
| 80 | 53 | *Parma microlepis* | microsatellite | [Curley & Gillings 2009](#_ENREF_33) | | | |
| 84 | 56 | *Phyllospora comosa* | microsatellite | [Coleman & Kelaher 2009](#_ENREF_29) | | | |
| 85 | 57 | *Macquaria colonorum* | mtDNA seq | [Shaddick *et al.* 2011](#_ENREF_84) | | | |
| 86 | 57 | *Macquaria colonorum* | microsatellite |  | | | |
| 87 | 58 | *Acropora tenuis* | microsatellite | [Underwood 2009](#_ENREF_99) | | | |
| 88 | 59 | *Haliotis rubra* | microsatellite | [Miller *et al.* 2009](#_ENREF_67) | | | |
| 90 | 61 | *Seriola lalandi* | microsatellite | [Miller *et al.* 2011](#_ENREF_68) | | | |
| 95 | 64 | *Cephalopholis argus* | mtDNA seq | [Gaither *et al.* 2011a](#_ENREF_42) | | | |
| 96 | 64 | *Cephalopholis argus* | nuclear seq |  | | | |
| 98 | 66 | *Pagrus auratus* | allozyme | [Sumpton *et al.* 2008](#_ENREF_94) | | | |
| 99 | 67 | *Microcosmus squamiger* | mtDNA seq | [Rius *et al.* 2008](#_ENREF_80) | | | |
| 100 | 68 | *Eudyptula minor* | microsatellite | [Overeem *et al.* 2008](#_ENREF_77) | | | |
| 102 | 69 | *Acanthochromis polyacanthus* | microsatellite | [Miller-Sims *et al.* 2008](#_ENREF_69) | | | |
| 103 | 70 | *Goniastrea favulus* | microsatellite | [Miller & Ayre 2008a](#_ENREF_65) | | | |
| 104 | 70 | *Platygyra daedalea* | microsatellite |  | | | |
| 105 | 71 | *Pocillopora damicornis* | allozyme | [Miller & Ayre 2008b](#_ENREF_66) | | | |
| 106 | 71 | *Goniastrea australensis* | allozyme |  | | | |
| 107 | 72 | *Aipysurus laevis* | microsatellite | [Lukoschek *et al.* 2008](#_ENREF_61) | | | |
| 117 | 76 | *Phyllospora comosa* | microsatellite | [Coleman *et al.* 2008](#_ENREF_27) | | | |
| 118 | 77 | *Delphinus delphis* | microsatellite | [Bilgmann *et al.* 2008](#_ENREF_14) | | | |
| 119 | 77 | *Delphinus delphis* | mtDNA seq |  | | | |
| 120 | 78 | *Acropora millepora* | microsatellite | [Souter *et al.* 2010](#_ENREF_90) | | | |
| 121 | 78 | *Acropora spathulata* | microsatellite | | | | |
| 123 | 80 | *Ecklonia radiata* | microsatellite | [Coleman *et al.* 2009](#_ENREF_28) | | | |
| 124 | 81 | *Haliotis rubra* | mtDNA RE | [Appleyard *et al.* 2009](#_ENREF_1) | | | |
| 125 | 81 | *Haliotis rubra* | microsatellite | | |  | |
| 128 | 83 | *Seriatopora hystrix* | microsatellite | [Underwood *et al.* 2007](#_ENREF_100) | | | |
| 129 | 84 | *Latris lineata* | mtDNA seq | [Tracey *et al.* 2007](#_ENREF_98) | | | |
| 130 | 85 | *Aipysurus laevis* | mtDNA seq | [Lukoschek *et al.* 2007](#_ENREF_62) | | | |
| 131 | 86 | *Pericharax heteroraphis* | nuclear seq | [Bentlage & Worheide 2007](#_ENREF_5) | | | |
| 132 | 87 | *Penaeus esculentus* | microsatellite | [Ward *et al.* 2006](#_ENREF_115) | | | |
| 133 | 88 | *Lumnitzera racemosa* | other | [Su *et al.* 2006](#_ENREF_92) | | | |
| 134 | 89 | *Acropora millepora* | allozyme | [Smith-Keune & van Oppen 2006](#_ENREF_88) | | | |
| 138 | 92 | *Acropora tenuis* | microsatellite | | | [Underwood *et al.* 2009](#_ENREF_101) | |
| 139 | 92 | *Seriatopora hystrix* | microsatellite | | | | |
| 142 | 94 | *Hyperoglyphe antarctica* | mtDNA seq | [Robinson *et al.* 2008](#_ENREF_82) | | | |
| 143 | 94 | *Seriolella brama* | mtDNA seq |  | | | |
| 148 | 97 | *Haliotis rubra* | microsatellite | [Temby *et al.* 2007](#_ENREF_95) | | | |
| 149 | 98 | *Anguilla australis* | microsatellite | [Shen & Tzeng 2007b](#_ENREF_86) | | | |
| 150 | 99 | *Chelonia mydas* | other | [Dethmers *et al.* 2006](#_ENREF_35) | | | |
| 151 | 100 | *Avicennia marina* | microsatellite | [Arnaud-Haond *et al.* 2006](#_ENREF_2) | | | |
| 152 | 101 | *Tursiops aduncas* | microsatellite | [Moller *et al.* 2007](#_ENREF_72) | | | |
| 153 | 101 | *Tursiops aduncas* | mtDNA seq |  | | | |
| 157 | 105 | *Stichopus chloronotus* | allozyme | [Uthicke *et al.* 1999](#_ENREF_105) | | | |
| 160 | 107 | *Catostylu mosaicus* | mtDNA seq | [Dawson 2005](#_ENREF_34) | | | |
| 161 | 107 | *Catostylu mosaicus* | nuclear seq |  | | | |
| 162 | 108 | *Patiriella exigua* | mtDNA seq | [Colgan *et al.* 2005](#_ENREF_30) | | | |
| 167 | 112 | *Avicennia marina* | allozyme | [Melville *et al.* 2004](#_ENREF_64) | | | |
| 168 | 113 | *Acanthopagrus butcheri* | allozyme | [Burridge *et al.* 2004](#_ENREF_22) | | | |
| 169 | 113 | *Acanthopagrus butcheri* | mtDNA RE |  | | | |
| 170 | 114 | *Arripis georgiana* | allozyme | [Ayvazian *et al.* 2004](#_ENREF_3) | | | |
| 176 | 116 | *Tursiops sp* | other | [Bilgmann *et al.* 2007](#_ENREF_15) | | | |
| 177 | 116 | *Tursiops sp* | microsatellite |  | | | |
| 192 | 122 | *Tursiops sp* | microsatellite | [Krutzen *et al.* 2004](#_ENREF_57) | | | |
| 193 | 122 | *Tursiops sp* | mtDNA seq |  | | | |
| 194 | 123 | *Lethrinus miniatus* | microsatellite | [van Herwerden *et al.* 2003](#_ENREF_108) | | | |
| 195 | 124 | *Holothuria nobilis* | mtDNA seq | [Uthicke & Benzie 2003](#_ENREF_103) | | | |
| 199 | 127 | *Sepia apama* | microsatellite | [Kassahn *et al.* 2003](#_ENREF_55) | | | |
| 201 | 129 | *Polynemus sheridani* | mtDNA seq | [Chenoweth & Hughes 2003](#_ENREF_25) | | | |
| 208 | 134 | *Penaeus monodon* | mtDNA RE | [Benzie *et al.* 2002](#_ENREF_7) | | | |
| 211 | 137 | *Acanthochromis polyacanthus* | mtDNA seq | [Planes *et al.* 2001](#_ENREF_79) | | | |
| 212 | 138 | *Seriola lalandi* | microsatellite | [Nugroho *et al.* 2001](#_ENREF_74) | | | |
| 213 | 138 | *Seriola lalandi* | mtDNA seq |  | | | |
| 216 | 141 | *Holothuria nobilis* | allozyme | [Uthicke & Benzie 2000](#_ENREF_102) | | | |
| 221 | 145 | *Penaeus monodon* | microsatellite | [Brooker *et al.* 2000](#_ENREF_20) | | | |
| 232 | 148 | *Scylla serrata* | mtDNA seq | [Gopurenko *et al.* 1999](#_ENREF_46) | | | |
| 234 | 150 | *Holothuria atra* | allozyme | [Uthicke *et al.* 1998](#_ENREF_104) | | | |
| 238 | 153 | *Lates calcarifer* | mtDNA seq | [Chenoweth *et al.* 1998](#_ENREF_26) | | | |
| 240 | 155 | *Linckia laevigata* | mtDNA RE | [Williams & Benzie 1997](#_ENREF_117) | | | |
| 274 | 174 | *Pocillopora damicornis* | allozyme | [Benzie *et al.* 1995](#_ENREF_8) | | | |
| 275 | 174 | *Acropora palifera* | allozyme |  | | | |
| 277 | 176 | *Anguilla reinhardtii* | microsatellite | [Shen & Tzeng 2007a](#_ENREF_85) | | | |
| 284 | 180 | *Scylla serrata* | mtDNA seq | [Gopurenko & Hughes 2002](#_ENREF_45) | | | |
| 285 | 181 | *Nemadactylus macropterus* | microsatellite | [Burridge & Smolenski 2003](#_ENREF_23) | | | |
| 286 | 182 | *Nemadactylus macropterus* | mtDNA RE | [Grewe *et al.* 1994](#_ENREF_47) | | | |
| 294 | 186 | *Trachurus declivis* | mtDNA RE | [Smolenski *et al.* 1994](#_ENREF_89) | | | |
| 300 | 191 | *Haliotis rubra* | mtDNA RE | [Conod *et al.* 2002](#_ENREF_32) | | | |
| 301 | 191 | *Haliotis rubra* | microsatellite | | |  | |
| 302 | 192 | *Pinctada imbricata* | allozyme | [Colgan & Ponder 2002](#_ENREF_31) | | | |
| 303 | 192 | *Pinctada albina* | allozyme |  | | | |
| 306 | 194 | *Rhombosolea tapirina* | allozyme | [van den Enden *et al.* 2000](#_ENREF_106) | | | |
| 309 | 197 | *Mustelus antarcticus* | allozyme | [Gardner & Ward 1998](#_ENREF_44) | | | |
| 329 | 209 | *Jasus edwardsii* | mtDNA RE | [Ovenden *et al.* 1992](#_ENREF_75) | | | |
| 333 | 212 | *Macquaria novemaculeata* | allozyme | [Jerry 1997](#_ENREF_54) | | | |
| 338 | 217 | *Haliotis laevigata* | microsatellite | [Maynard *et al.* 2004](#_ENREF_63) | | |  |
| 342 | 221 | *Caloglossa leprieurii* | other | [Zuccarello *et al.* 2000](#_ENREF_120) | | | |
| 346 | 223 | *Lates calcarifer* | mtDNA seq | [Doupe *et al.* 1999](#_ENREF_37) | | | |
| 352 | 224 | *Allocyttus verrucosus* | mtDNA RE | [Ward *et al.* 1998](#_ENREF_114) | | | |
| 354 | 226 | *Neocyttus rhomboidalis* | mtDNA RE | [Elliott *et al.* 1998](#_ENREF_39) | | | |
| 355 | 226 | *Neocyttus rhomboidalis* | allozyme |  | | | |
| 373 | 64 | *Cephalopholis argus* | nuclear seq | [Gaither *et al.* 2011a](#_ENREF_42) | | | |
| 374 | 239 | *Pinctada maxima* | mtDNA RE | [Benzie *et al.* 2003](#_ENREF_9) | | | |
| 376 | 241 | *Pristipomoides filamentosus* | mtDNA seq | [Gaither *et al.* 2011b](#_ENREF_43) | | | |
| 377 | 241 | *Pristipomoides filamentosus* | microsatellite | | |  | |
| 378 | 242 | *Naso brevirostris* | mtDNA seq | [Horne *et al.* 2008](#_ENREF_52) | | | |
| 379 | 243 | *Naso vlamingii* | mtDNA seq | [Klanten *et al.* 2007](#_ENREF_56) | | | |
| 380 | 244 | *Haliotis asinina* | mtDNA seq | [Imron *et al.* 2007](#_ENREF_53) | | | |
| 381 | 245 | *Haliotis rubra* | microsatellite | [Li *et al.* 2006](#_ENREF_59) | | | |
| 382 | 245 | *Haliotis rubra* | mtDNA RE |  | | | |
| 385 | 247 | *Haliotis coccoradiata* | microsatellite | [Piggott *et al.* 2008](#_ENREF_78) | | | |
| 390 | 251 | *Actinia tenebarosa* | allozyme | [Sherman *et al.* 2008](#_ENREF_87) | | | |
| 403 | 78 | *Acropora pulchra* | microsatellite | [Souter *et al.* 2010](#_ENREF_90) | | | |

**References**

Appleyard SA, Carr NA, Elliott NG (2009) Molecular analyses indicate homogenous structure of abalone across morphologically different *Haliotis rubra* collections in South Australia. *Journal of Shellfish Research* **28**, 609-616.

Arnaud-Haond S, Teixeira S, Massa SI*, et al.* (2006) Genetic structure at range edge: low diversity and high inbreeding in Southeast Asian mangrove (Avicennia marina) populations. *Molecular Ecology* **15**, 3515-3525.

Ayvazian SG, Bastow TP, Edmonds JS, How J, Nowara GB (2004) Stock structure of Australian herring (*Arripis georgiana*) in southwestern Australia. *Fisheries Research* **67**, 39-53.

Banks SC, Ling SD, Johnson CR*, et al.* (2010) Genetic structure of a recent climate change-driven range extension. *Molecular Ecology* **19**, 2011-2024.

Bentlage B, Worheide G (2007) Low genetic structuring among *Pericharax heteroraphis* (Porifera : Calcarea) populations from the Great Barrier Reef (Australia), revealed by analysis of nrDNA and nuclear intron sequences. *Coral Reefs* **26**, 807-816.

Benzie JAH (1991) Genetic relatedness of foraminiferan (*Marginopora vertebralis*) populations from reefs in the Western Coral Sea and Great Barrier Reef. *Coral Reefs* **10**, 29-36.

Benzie JAH, Ballment E, Forbes AT*, et al.* (2002) Mitochondrial DNA variation in Indo-Pacific populations of the giant tiger prawn, *Penaeus monodon*. *Molecular Ecology* **11**, 2553-2569.

Benzie JAH, Haskell A, Lehman H (1995) Variation in the genetic composition of coral (*Pocillopora damicornis* and *Acropora palifera*) populations from different reef habitats. *Marine Biology* **121**, 731-739.

Benzie JAH, Smith C, Sugama K (2003) Mitochondrial DNA reveals genetic differentiation between Australian and Indonesian pearl oyster *Pinctada maxima* (Jameson 1901) populations. *Journal of Shellfish Research* **22**, 781-787.

Benzie JAH, Smith-Keune C (2006) Microsatellite variation in Australian and Indonesian pearl oyster *Pinctada maxima* populations. *Marine Ecology Progress Series* **314**, 197-211.

Benzie JAH, Stoddart JA (1992) Genetic structure of crown-of-thorns starfish (*Acanthaster planci*) in Australia. *Marine Biology* **112**, 631-639.

Berry O, England P, Fairclough D, Jackson G, Greenwood J (2012a) Microsatellite DNA analysis and hydrodynamic modelling reveal the extent of larval transport and gene flow between management zones in an exploited marine fish (*Glaucosoma hebraicum*). *Fisheries Oceanography* **21**, 243-254.

Berry O, England P, Marriott RJ, Burridge CP, Newman SJ (2012b) Understanding age-specific dispersal in fishes through hydrodynamic modelling, genetic simulations and microsatellite DNA analysis. *Molecular Ecology* **21**, 2145-2159.

Bilgmann K, Moller LM, Harcourt RG, Gales R, Beheregaray LB (2008) Common dolphins subject to fisheries impacts in Southern Australia are genetically differentiated: implications for conservation. *Animal Conservation* **11**, 518-528.

Bilgmann K, Moller LM, Harcourt RG, Gibbs SE, Beheregaray LB (2007) Genetic differentiation in bottlenose dolphins from South Australia: association with local oceanography and coastal geography. *Marine Ecology-Progress Series* **341**, 265-276.

Binks RM, Evans JP, Prince J, Kennington WJ (2011) Fine-scale patterns of genetic divergence within and between morphologically variable subspecies of the sea urchin *Heliocidaris erythrogramma* (Echinometridae). *Biological Journal of the Linnean Society* **103**, 578-592.

Blower DC, Pandolfi JM, Bruce BD, Gomez-Cabrera MD, Ovenden JR (2012) Population genetics of Australian white sharks reveals fine-scale spatial structure, transoceanic dispersal events and low effective population sizes. *Marine Ecology Progress Series* **455**, 229-244.

Bongaerts P, Riginos C, Ridgway T*, et al.* (2010) Genetic divergence across habitats in the widespread coral *Seriatopora hystrix* and its associated symbiodinium. *Plos One* **5**.

Broderick D, Ovenden JR, Buckworth RC*, et al.* (2011) Genetic population structure of grey mackerel *Scomberomorus semifasciatus* in northern Australia. *Journal of Fish Biology* **79**, 633-661.

Brooker AL, Benzie JAH, Blair D, Versini JJ (2000) Population structure of the giant tiger prawn *Penaeus monodon* in Australian waters, determined using microsatellite markers. *Marine Biology* **136**, 149-157.

Brown LD (1991) Genetic variation and population structure in the blacklip abalone, *Haliotis rubra*. *Australian Journal of Marine and Freshwater Research* **42**, 77-90.

Burridge CP, Hurt AC, Farrington LW, Coutin PC, Austin CM (2004) Stepping stone gene flow in an estuarine-dwelling sparid from south-east Australia. *Journal of Fish Biology* **64**, 805-819.

Burridge CP, Smolenski AJ (2003) Lack of genetic divergence found with microsatellite DNA markers in the tarakihi *Nemadactylus macropterus*. *New Zealand Journal of Marine and Freshwater Research* **37**, 223-230.

Carroll E, Patenaude N, Alexander A*, et al.* (2011) Population structure and individual movement of southern right whales around New Zealand and Australia. *Marine Ecology Progress Series* **432**, 257.

Chenoweth SF, Hughes JM (2003) Oceanic interchange and nonequilibrium population structure in the estuarine dependent Indo-Pacific tasselfish, *Polynemus sheridani*. *Molecular Ecology* **12**, 2387-2397.

Chenoweth SF, Hughes JM, Keenan CP, Lavery S (1998) Concordance between dispersal and mitochondrial gene flow: isolation by distance in a tropical teleost, *Lates calcarifer* (Australian barramundi). *Heredity* **80**, 187-197.

Coleman MA, Dolman G, Kelaher BP, Steinberg PD (2008) Characterisation of microsatellite loci in the subtidal habitat-forming alga, *Phyllospora comosa* (Phaeophyceae, Fucales). *Conservation Genetics* **9**, 1015-1017.

Coleman MA, Gillanders BM, Connell SD (2009) Dispersal and gene flow in the habitat-forming kelp, *Ecklonia radiata*: relative degrees of isolation across an east-west coastline. *Marine and Freshwater Research* **60**, 802-809.

Coleman MA, Kelaher BP (2009) Connectivity among fragmented populations of a habitat-forming alga, *Phyllospora comosa* (Phaeophyceae, Fucales) on an urbanised coast. *Marine Ecology Progress Series* **381**, 63-70.

Colgan DJ, Byrne M, Rickard E, Castro LR (2005) Limited nucleotide divergence over large spatial scales in the asterinid sea star *Patiriella exigua*. *Marine Biology* **146**, 263-270.

Colgan DJ, Ponder WF (2002) Genetic discrimination of morphologically similar, sympatric species of pearl oysters (Mollusca: Bivalvia: Pinctada) in eastern Australia. *Marine and Freshwater Research* **53**, 697-709.

Conod N, Bartlett JP, Evans BS, Elliott NG (2002) Comparison of mitochondrial and nuclear DNA analyses of population structure in the blacklip abalone *Haliotis rubra* Leach. *Marine and Freshwater Research* **53**, 711-718.

Curley BG, Gillings MR (2009) Population connectivity in the temperate damselfish *Parma microlepis*: analyses of genetic structure across multiple spatial scales. *Marine Biology* **156**, 381-393.

Dawson MN (2005) Incipient speciation of *Catostylus mosaicus* (Scyphozoa, Rhizostomeae, Catostylidae), comparative phylogeography and biogeography in south-east Australia. *Journal of Biogeography* **32**, 515-533.

Dethmers KEM, Broderick D, Moritz C*, et al.* (2006) The genetic structure of Australasian green turtles (*Chelonia mydas*): exploring the geographical scale of genetic exchange. *Molecular Ecology* **15**, 3931-3946.

Doubleday ZA, Semmens JM, Smolenski AJ, Shaw PW (2009) Microsatellite DNA markers and morphometrics reveal a complex population structure in a merobenthic octopus species (*Octopus maorum*) in south-east Australia and New Zealand. *Marine Biology* **156**, 1183-1192.

Doupe RG, Horwitz P, Lymbery AJ (1999) Mitochondrial genealogy of Western Australian barramundi: applications of inbreeding coefficients and coalescent analysis for separating temporal population processes. *Journal of Fish Biology* **54**, 1197-1209.

Dudgeon CL, Broderick D, Ovenden JR (2009) IUCN classification zones concord with, but underestimate, the population genetic structure of the zebra shark *Stegostoma fasciatum* in the Indo-West Pacific. *Molecular Ecology* **18**, 248-261.

Elliott NG, Lowry PS, Grewe PM*, et al.* (1998) Genetic evidence for depth-and spatially separated stocks of the deep-water spikey oreo in Australasian waters. *Journal of Fish Biology* **52**, 796-816.

Evans RD, van Herwerden L, Russ GR, Frisch AJ (2010) Strong genetic but not spatial subdivision of two reef fish species targeted by fishers on the Great Barrier Reef. *Fisheries Research* **102**, 16-25.

Fauvelot C, Borsa P (2011) Patterns of genetic isolation in a widely distributed pelagic fish, the narrow-barred Spanish mackerel (*Scomberomorus commerson*). *Biological Journal of the Linnean Society* **104**, 886-902.

Gaither MR, Bowen BW, Bordenave TR*, et al.* (2011a) Phylogeography of the reef fish *Cephalopholis argus* (Epinephelidae) indicates Pleistocene isolation across the indo-pacific barrier with contemporary overlap in the coral triangle. *Bmc Evolutionary Biology* **11**.

Gaither MR, Jones SA, Kelley C*, et al.* (2011b) High Connectivity in the Deepwater Snapper *Pristipomoides filamentosus* (Lutjanidae) across the Indo-Pacific with Isolation of the Hawaiian Archipelago. *Plos One* **6**.

Gardner MG, Ward RD (1998) Population structure of the Australian gummy shark (*Mustelus antarcticus* Gunther) inferred from allozymes, mitochondrial DNA and vertebrae counts. *Marine and Freshwater Research* **49**, 733-745.

Gopurenko D, Hughes JM (2002) Regional patterns of genetic structure among Australian populations of the mud crab, *Scylla serrata* (Crustacea : Decapoda): evidence from mitochondrial DNA. *Marine and Freshwater Research* **53**, 849-857.

Gopurenko D, Hughes JM, Keenan CP (1999) Mitochondrial DNA evidence for rapid colonisation of the Indo-West Pacific by the mudcrab *Scylla serrata*. *Marine Biology* **134**, 227-233.

Grewe PM, Smolenski AJ, Ward RD (1994) Mitochondrial DNA diversity in Jackass Morwong (*Nemadactylus macropterus*, Teleostei) from Australian and New Zealand waters. *Canadian Journal of Fisheries and Aquatic Sciences* **51**, 1101-1109.

Haig JA, Connolly RM, Hughes JM (2010) Little shrimp left on the shelf: the roles that sea-level change, ocean currents and continental shelf width play in the genetic connectivity of a seagrass-associated species. *Journal of Biogeography* **37**, 1570-1583.

Hammond LS, Poiner IR (1984) Genetic structure of three populations of the 'living fossil' brachiopod *Lingula* from Queensland, Australia. *Lethaia* **17**, 139-143.

Horne JB, Momigliano P, Welch DJ, Newman SJ, van Herwerden L (2011) Limited ecological population connectivity suggests low demands on self-recruitment in a tropical inshore marine fish (*Eleutheronema tetradactylum*: Polynemidae). *Molecular Ecology* **20**, 2291-2306.

Horne JB, Momigliano P, Welch DJ, Newman SJ, van Herwerden L (2012) Searching for common threads in threadfins: phylogeography of Australian polynemids in space and time. *Marine Ecology-Progress Series* **449**, 263-276.

Horne JB, van Herwerden L, Choat JH, Robertson DR (2008) High population connectivity across the Indo-Pacific: congruent lack of phylogeographic structure in three reef fish congeners. *Molecular Phylogenetics and Evolution* **49**, 629-638.

Imron, Jeffrey B, Hale P, Degnan BM, Degnan SM (2007) Pleistocene isolation and recent gene flow in *Haliotis asinina*, an Indo-Pacific vetigastropod with limited dispersal capacity. *Molecular Ecology* **16**, 289-304.

Jerry DR (1997) Population genetic structure of the catadromous Australian bass from throughout its range. *Journal of Fish Biology* **51**, 909-920.

Kassahn KS, Donnellan SC, Fowler AJ*, et al.* (2003) Molecular and morphological analyses of the cuttlefish *Sepia apama* indicate a complex population structure. *Marine Biology* **143**, 947-962.

Klanten OS, Choat JH, van Herwerden L (2007) Extreme genetic diversity and temporal rather than spatial partitioning in a widely distributed coral reef fish. *Marine Biology* **150**, 659-670.

Krutzen M, Sherwin WB, Berggren P, Gales N (2004) Population structure in an inshore cetacean revealed by microsatellite and mtDNA analysis: bottlenose dolphins (*Tursiops* sp.) in Shark Bay, Western Australia. *Marine Mammal Science* **20**, 28-47.

Lancaster ML, Arnould JPY, Kirkwood R (2010) Genetic status of an endemic marine mammal, the Australian fur seal, following historical harvesting. *Animal Conservation* **13**, 247-255.

Li Z, Appleyard SA, Elliott NG (2006) Population structure of *Haliotis rubra* from South Australia inferred from nuclear and mtDNA analyses. *Acta Oceanologica Sinica* **25**, 99-112.

Lowther AD, Harcourt RG, Goldsworthy SD, Stow A (2012) Population structure of adult female Australian sea lions is driven by fine-scale foraging site fidelity. *Animal Behaviour* **83**, 691-701.

Lukoschek V, Waycott M, Keogh JS (2008) Relative information content of polymorphic microsatellites and mitochondrial DNA for inferring dispersal and population genetic structure in the olive sea snake, *Aipysurus laevis*. *Molecular Ecology* **17**, 3062-3077.

Lukoschek V, Waycott M, Marsh H (2007) Phylogeography of the olive sea snake, *Aipysurus laevis (*Hydrophiinae) indicates Pleistocene range expansion around northern Australia but low contemporary gene flow. *Molecular Ecology* **16**, 3406-3422.

Maynard BT, Hanna PJ, Benzie JAH (2004) Microsatellite DNA analysis of southeast Australian *Haliotis laevigata* (Donovan) populations - Implications for Ranching in Port Phillip Bay. *Journal of Shellfish Research* **23**, 1195-1200.

Melville F, Burchett M, Pulkownik A (2004) Genetic variation among age-classes of the mangrove *Avicennia marina* in clean and contaminated sediments. *Marine Pollution Bulletin* **49**, 695-703.

Miller KJ, Ayre DJ (2008a) Population structure is not a simple function of reproductive mode and larval type: insights from tropical corals. *Journal of Animal Ecology* **77**, 713-724.

Miller KJ, Ayre DJ (2008b) Protection of genetic diversity and maintenance of connectivity among reef corals within marine protected areas. *Conservation Biology* **22**, 1245-1254.

Miller KJ, Maynard BT, Mundy CN (2009) Genetic diversity and gene flow in collapsed and healthy abalone fisheries. *Molecular Ecology* **18**, 200-211.

Miller PA, Fitch AJ, Gardner M, Hutson KS, Mair G (2011) Genetic population structure of Yellowtail Kingfish (*Seriola lalandi*) in temperate Australasian waters inferred from microsatellite markers and mitochondrial DNA. *Aquaculture* **319**, 328-336.

Miller-Sims VC, Gerlach G, Kingsford MJ, Atema J (2008) Dispersal in the spiny damselfish, *Acanthochromis polyacanthus*, a coral reef fish species without a larval pelagic stage. *Molecular Ecology* **17**, 5036-5048.

Mirams AGK, Treml EA, Shields JL, Liggins L, Riginos C (2011) Vicariance and dispersal across an intermittent barrier: population genetic structure of marine animals across the Torres Strait land bridge. *Coral Reefs* **30**, 937-949.

Moller L, Valdez FP, Allen S*, et al.* (2011) Fine-scale genetic structure in short-beaked common dolphins (*Delphinus delphis*) along the East Australian Current. *Marine Biology* **158**, 113-126.

Moller LM, Wiszniewski J, Allen SJ, Beheregaray LB (2007) Habitat type promotes rapid and extremely localised genetic differentiation in dolphins. *Marine and Freshwater Research* **58**, 640-648.

Nash WJ, Goddard M, Lucas JS (1988) Population genetic studies of the crown-of-thorns starfish, *Acanthaster planci* (L.), in the Great Barrier Reef region. *Coral Reefs* **7**, 11-18.

Nugroho E, Ferrell DJ, Smith P, Taniguchi N (2001) Genetic divergence of kingfish from Japan, Australia and New Zealand inferred by microsatellite DNA and mitochondrial DNA control region markers. *Fisheries Science (Tokyo)* **67**, 843-850.

Ovenden JR, Brasher DJ, White RWG (1992) Mitochondrial DNA analyses of red rock lobster *Jasus edwardsii* supports an apparent absence of population subdivision throughout Australasia. *Marine Biology (Berlin)* **112**, 319-326.

Ovenden JR, Morgan JAT, Street R*, et al.* (2011) Negligible evidence for regional genetic population structure for two shark species *Rhizoprionodon acutus* (Ruppell, 1837) and *Sphyrna lewini* (Griffith & Smith, 1834) with contrasting biology. *Marine Biology (Berlin)* **158**, 1497-1509.

Overeem RL, Peucker AJ, Austin CM, Dann P, Burridge CP (2008) Contrasting genetic structuring between colonies of the World's smallest penguin, *Eudyptula minor* (Aves : Spheniscidae). *Conservation Genetics* **9**, 893-905.

Piggott MP, Banks SC, Tung P, Beheregaray LB (2008) Genetic evidence for different scales of connectivity in a marine mollusc. *Marine Ecology Progress Series* **365**, 127-136.

Planes S, Doherty PJ, Bernardi G (2001) Strong genetic divergence among populations of a marine fish with limited dispersal, *Acanthochromis polyacanthus*, within the Great Barrier Reef and the Coral Sea. *Evolution* **55**, 2263-2273.

Rius M, Pascual M, Turon X (2008) Phylogeography of the widespread marine invader Microcosmus squamiger(Ascidiacea) reveals high genetic diversity of introduced populations and non-independent colonizations. *Diversity and Distributions* **14**, 818-828.

Roberts DG, Ayre DJ (2010) Panmictic population structure in the migratory marine sparid *Acanthopagrus australis* despite its close association with estuaries. *Marine Ecology Progress Series* **412**, 223-230.

Robinson N, Skinner A, Sethuraman L*, et al.* (2008) Genetic stock structure of blue-eye trevalla (*Hyperoglyphe antarctica*) and warehous (*Seriolella brama* and *Seriolella punctata*) in south-eastern Australian waters. *Marine and Freshwater Research* **59**, 502-514.

Schmidt DJ, Crook DA, O'Connor JP, Hughes JM (2011) Genetic analysis of threatened Australian grayling *Prototroctes maraena* suggests recruitment to coastal rivers from an unstructured marine larval source population. *Journal of Fish Biology* **78**, 98-111.

Shaddick K, Gilligan DM, Burridge CP*, et al.* (2011) Historic divergence with contemporary connectivity in a catadromous fish, the estuary perch (*Macquaria colonorum*). *Canadian Journal of Fisheries and Aquatic Sciences* **68**, 304-318.

Shen K-N, Tzeng W-N (2007a) Population genetic structure of the year-round spawning tropical eel, *Anguilla reinhardti*, in Australia. *Zoological Studies* **46**, 441-453.

Shen KN, Tzeng WN (2007b) Genetic differentiation among populations of the shortfinned eel *Anguilla australis* from East Australia and New Zealand. *Journal of Fish Biology* **70**, 177-190.

Sherman CDH, Hunt A, Ayre DJ (2008) Is life history a barrier to dispersal? Contrasting patterns of genetic differentiation along an oceanographically complex coast. *Biological Journal of the Linnean Society* **95**, 106-116.

Smith-Keune C, van Oppen M (2006) Genetic structure of a reef-building coral from thermally distinct environments on the Great Barrier Reef. *Coral Reefs* **25**, 493-502.

Smolenski AJ, Ovenden JR, White RWG (1994) Preliminary investigation of mitochondrial DNA variation in Jack Mackerel (*Trachurus declivis*, Carangidae) from south-eastern Australian waters. *Australian Journal of Marine and Freshwater Research* **45**, 495-505.

Souter P, Willis BL, Bay LK*, et al.* (2010) Location and disturbance affect population genetic structure in four coral species of the genus *Acropora* on the Great Barrier Reef. *Marine Ecology-Progress Series* **416**, 35-45.

Stoddart JA (1984) Genetical structure within populations of the coral *Pocillopora damicornis*. *Marine Biology* **81**, 19-30.

Su GH, Huang YL, Tan FX*, et al.* (2006) Genetic variation in *Lumnitzera racemosa*, a mangrove species from the Indo-West Pacific. *Aquatic Botany* **84**, 341-346.

Sulaiman ZH, Ovenden JR (2010) Population genetic evidence for the east-west division of the narrow-barred Spanish mackerel (*Scomberomorus commerson*, Perciformes: Teleostei) along Wallace's Line. *Biodiversity and Conservation* **19**, 563-574.

Sumpton WD, Ovenden JR, Keenan CP, Street R (2008) Evidence for a stock discontinuity of snapper (*Pagrus auratus*) on the east coast of Australia. *Fisheries Research* **94**, 92-98.

Temby N, Miller K, Mundy C (2007) Evidence of genetic subdivision among populations of blacklip abalone (*Haliotis rubra* Leach) in Tasmania. *Marine and Freshwater Research* **58**, 733-742.

Tillett BJ, Meekan MG, Broderick D*, et al.* (2012a) Pleistocene isolation, secondary introgression and restricted contemporary gene flow in the pig-eye shark, *Carcharhinus amboinensis* across northern Australia. *Conservation Genetics* **13**, 99-115.

Tillett BJ, Meekan MG, Field IC, Thorburn DC, Ovenden JR (2012b) Evidence for reproductive philopatry in the bull shark *Carcharhinus leucas*. *Journal of Fish Biology* **80**, 2140-2158.

Tracey SR, Smolenski A, Lyle JM (2007) Genetic structuring of *Latris lineata* at localized and transoceanic scales. *Marine Biology (Berlin)* **152**, 119-128.

Underwood JN (2009) Genetic diversity and divergence among coastal and offshore reefs in a hard coral depend on geographic discontinuity and oceanic currents. *Evolutionary Applications* **2**, 222-233.

Underwood JN, Smith LD, Van Oppen MJH, Gilmour JP (2007) Multiple scales of genetic connectivity in a brooding coral on isolated reefs following catastrophic bleaching. *Molecular Ecology* **16**, 771-784.

Underwood JN, Smith LD, van Oppen MJH, Gilmour JP (2009) Ecologically relevant dispersal of corals on isolated reefs: implications for managing resilience. *Ecological Applications* **19**, 18-29.

Uthicke S, Benzie JAH (2000) Allozyme electrophoresis indicates high gene flow between populations of *Holothuria (Microthele) nobilis* (Holothuroidea : Aspidochirotida) on the Great Barrier Reef. *Marine Biology* **137**, 819-825.

Uthicke S, Benzie JAH (2003) Gene flow and population history in high dispersal marine invertebrates: mitochondrial DNA analysis of *Holothuria nobilis (*Echinodermata : Holothuroidea) populations from the Indo-Pacific. *Molecular Ecology* **12**, 2635-2648.

Uthicke S, Benzie JAH, Ballment E (1998) Genetic structure of fissiparous populations of *Holothuria (Halodeima) atra* on the Great Barrier Reef. *Marine Biology* **132**, 141-151.

Uthicke S, Benzie JAH, Ballment E (1999) Population genetics of the fissiparous holothurian *Stichopus chloronotus* (Aspidochirotida) on the Great Barrier Reef, Australia. *Coral Reefs* **18**, 123-132.

van den Enden T, White RWG, Elliott NG (2000) Genetic variation in the greenback flounder *Rhombosolea tapirina* Gunther (Teleostei, Pleuronectidae) and the implications for aquaculture. *Marine and Freshwater Research* **51**, 23-33.

van Herwerden L, Aspden WJ, Newman SJ*, et al.* (2009a) A comparison of the population genetics of *Lethrinus miniatus* and *Lutjanus sebae* from the east and west coasts of Australia: Evidence for panmixia and isolation. *Fisheries Research* **100**, 148-155.

van Herwerden L, Benzie J, Davies C (2003) Microsatellite variation and population genetic structure of the red throat emperor on the Great Barrier Reef. *Journal of Fish Biology* **62**, 987-999.

van Herwerden L, Choat JH, Dudgeon CL*, et al.* (2006) Contrasting patterns of genetic structure in two species of the coral trout *Plectropomus* (Serranidae) from east and west Australia: Introgressive hybridisation or ancestral polymorphisms. *Molecular Phylogenetics and Evolution* **41**, 420-435.

van Herwerden L, Choat JH, Newman SJ, Leray M, Hillersoy G (2009b) Complex patterns of population structure and recruitment of *Plectropomus leopardus* (Pisces: Epinephelidae) in the Indo-West Pacific: implications for fisheries management. *Marine Biology* **156**, 1595-1607.

van Oppen MJH, Bongaerts P, Underwood JN, Peplow LM, Cooper TF (2011a) The role of deep reefs in shallow reef recovery: an assessment of vertical connectivity in a brooding coral from west and east Australia. *Molecular Ecology* **20**, 1647-1660.

van Oppen MJH, Peplow LM, Kininmonth S, Berkelmans R (2011b) Historical and contemporary factors shape the population genetic structure of the broadcast spawning coral, *Acropora millepora*, on the Great Barrier Reef. *Molecular Ecology* **20**, 4899-4914.

Veilleux HD, van Herwerden L, Evans RD, Travers MJ, Newman S (2011) Strong genetic subdivision generates high genetic variability among eastern and western Australian populations of *Lutjanus carponotatus* (Richardson). *Fisheries Research* **108**, 74-80.

Ward RD, Elliott NG, Grewe PM*, et al.* (1998) Allozyme and mitochondrial DNA variation in three species of oreos (Teleostei : Oreosomatidae) from Australasian waters. *New Zealand Journal of Marine and Freshwater Research* **32**, 233-245.

Ward RD, Ovenden JR, Meadows JRS, Grewe PM, Lehnert SA (2006) Population genetic structure of the brown tiger prawn, *Penaeus esculentus*, in tropical northern Australia. *Marine Biology* **148**, 599-607.

Whitney NM, Robbins WD, Schultz JK, Bowen BW, Holland KN (2012) Oceanic dispersal in a sedentary reef shark (*Triaenodon obesus*): genetic evidence for extensive connectivity without a pelagic larval stage. *Journal of Biogeography* **39**, 1144-1156.

Williams ST, Benzie JAH (1997) Indo-West Pacific patterns of genetic differentiation in the high-dispersal starfish *Linckia laevigata*. *Molecular Ecology* **6**, 559-573.

Wiszniewski J, Beheregaray LB, Allen SJ, Moller L (2010) Environmental and social influences on the genetic structure of bottlenose dolphins (*Tursiops aduncus*) in Southeastern Australia. *Conservation Genetics* **11**, 1405-1419.

Yasuda N, Nagai S, Hamaguchi M*, et al.* (2009) Gene flow of *Acanthaster planci* (L.) in relation to ocean currents revealed by microsatellite analysis. *Molecular Ecology* **18**, 1574-1590.

Zuccarello GC, Bartlett J, Yeates PH (2000) Differentiation of *Caloglossa leprieurii* (Rhodophyta) populations in northern and eastern Australia using plastid haplotypes. *European Journal of Phycology* **35**, 357-363.
