# Supplementary material for: Population Genetic Diversity in the Australian ‘Seascape’: A Bioregion Approach
Source: PLoS One. 2015 Sep 16;10(9):e0136275. doi: 10.1371/journal.pone.0136275 (PMC4574161; doi:10.1371/journal.pone.0136275)
Supplement: S2 Table — (DOCX) [file pone.0136275.s007.docx]

**S2 Table**. Results from all models for both unstandardised (H) and standardized (Z) population genetic diversity measures. Explanatory variables are as follows: S = Species, M = genetic marker, L = latitude, L2 = latitude squared, R = IMCRA region. DIC is the deviance information criterion, and delta represents the difference of that models DIC from the best model. PPP is the p value from our Posterior predictive tests, and is based on the discrepancy between values ‘observed’ from the data and those generated from random sampling of the output files of the model (expected). Graphs represent the PPP results for the best DIC (S Z) and best PPP model (SnR Z) and the best model for both DIC and PPP (SMnLL2R H). The red line indicates the observed discrepancy statistic relative to those datasets generated by the model in grey.

|  | Explanatory vars | DIC | delta | PPP |
| --- | --- | --- | --- | --- |
|  | Z |  |  |  |
| * | S | 2674.8 | 0.0 | 1.00 |
|  | S + L | 2676.1 | 1.2 | 1.00 |
|  | S + L2 | 2678.9 | 4.0 | 1.00 |
|  | S + R | 2694.5 | 19.7 | 1.00 |
|  | S + L + R | 2698.39 | 23.5 | 1.00 |
|  | S + L + L2+ R | 2697.7 | 22.8 | 1.00 |
|  | S + n | 2676.3 | 1.4 | 0.00 |
|  | S + n + L | 2678.5 | 3.6 | 0.00 |
|  | S + n + L + L2 | 2680.9 | 6.0 | 0.00 |
| * | S + n + R | 2676.2 | 1.3 | 0.00 |
|  | S + n + L + R | 2677.1 | 2.3 | 0.00 |
|  | S + n + L + L2 + R | 2680.2 | 5.3 | 0.00 |
|  | H |  |  |  |
|  | S + M | -659.4 | 56.1 | 0.00 |
|  | S + M + L | -676.8 | 38.7 | 0.99 |
|  | S + M + L + L2 | -688.5 | 27.0 | 0.00 |
|  | S + M + R | -695.9 | 19.6 | 0.00 |
|  | S + M + L + R | -710.2 | 5.3 | 0.88 |
|  | S + M + L + L2 + R | -711.9 | 3.6 | 0.17 |
|  | S + M + n | -661.8 | 53.7 | 0.00 |
|  | S + M + n + L | -681.2 | 34.3 | 0.99 |
|  | S + M + n + L + L2 | -692.1 | 23.4 | 0.03 |
|  | S + M + n + R | -697.2 | 18.3 | 0.00 |
|  | S + M + n + L + R | -711.8 | 3.7 | 0.91 |
| ** | S + M + n + L + L2 + R | -715.5 | 0.0 | 0.36 |
